# An MST4‐p*β*‐Catenin^Thr40^ Signaling Axis Controls Intestinal Stem Cell and Tumorigenesis

**DOI:** 10.1002/advs.202004850

**Published:** 2021-07-08

**Authors:** Hui Zhang, Moubin Lin, Chao Dong, Yang Tang, Liwei An, Junyi Ju, Fuping Wen, Fan Chen, Meng Wang, Wenjia Wang, Min Chen, Yun Zhao, Jixi Li, Steven X. Hou, Xinhua Lin, Lulu Hu, Wenbo Bu, Dianqing Wu, Lin Li, Shi Jiao, Zhaocai Zhou

**Affiliations:** ^1^ State Key Laboratory of Molecular Biology CAS Center for Excellence in Molecular Cell Science Shanghai Institute of Biochemistry and Cell Biology Chinese Academy of Sciences University of Chinese Academy of Sciences Shanghai 200031 China; ^2^ State Key Laboratory of Genetic Engineering Department of Cell and Developmental Biology School of Life Sciences Zhongshan Hospital Fudan University Shanghai 200438 China; ^3^ Department of General Surgery Yangpu Hospital Tongji University School of Medicine Shanghai 200090 China; ^4^ Department of the Second Medical Oncology The 3rd Affiliated Hospital of Kunming Medical University Yunnan Tumor Hospital Kunming 650118 China; ^5^ Department of Medical Ultrasound Tongji University Cancer Center Shanghai Tenth People's Hospital School of Medicine Tongji University Shanghai 200072 China; ^6^ Fudan University Shanghai Cancer Center Institutes of Biomedical Sciences State Key Laboratory of Genetic Engineering and Shanghai Key Laboratory of Medical Epigenetics Shanghai Medical College of Fudan University Shanghai 200032 China; ^7^ Department of Materials Science Fudan University Shanghai 200433 China; ^8^ Department of Pharmacology Yale School of Medicine New Haven CT 06520 USA

**Keywords:** cancer stem cells, colorectal cancer, intestinal stem cells, MST4‐p*β*‐catenin^Thr40^ signaling axis, targeted therapy

## Abstract

Elevated Wnt/*β*‐catenin signaling has been commonly associated with tumorigenesis especially colorectal cancer (CRC). Here, an MST4‐p*β*‐catenin^Thr40^ signaling axis essential for intestinal stem cell (ISC) homeostasis and CRC development is uncovered. In response to Wnt3a stimulation, the kinase MST4 directly phosphorylates *β*‐catenin at Thr40 to block its Ser33 phosphorylation by GSK3*β*. Thus, MST4 mediates an active process that prevents *β*‐catenin from binding to and being degraded by *β*‐TrCP, leading to accumulation and full activation of *β*‐catenin. Depletion of MST4 causes loss of ISCs and inhibits CRC growth. Mice bearing either MST4^T178E^ mutation with constitutive kinase activity or *β*‐catenin^T40D^ mutation mimicking MST4‐mediated phosphorylation show overly increased ISCs/CSCs and exacerbates CRC. Furthermore, the MST4‐p*β*‐catenin^Thr40^ axis is upregulated and correlated with poor prognosis of human CRC. Collectively, this work establishes a previously undefined machinery for *β*‐catenin activation, and further reveals its function in stem cell and tumor biology, opening new opportunities for targeted therapy of CRC.

## Graphical abstract

A novel MST4‐p*β*‐catenin^Thr40^ signaling axis essential for maintaining intestinal stem cell (ISC) homeostasis is identified. In response to Wnt signal, MST4 directly phosphorylates *β*‐catenin at Thr40 to actively block the GSK3*β*/*β*‐TrCP‐mediated phosphorylation/degradation of *β*‐catenin. Genetic activation of the MST4‐p*β*‐catenin^Thr40^ axis exacerbates ISC‐driven colorectal cancer (CRC); and hyperactivation of this axis is clinically correlated with poor prognosis.

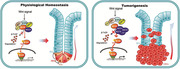

## 1 Introduction

To maintain tissue integrity and homeostasis, the intestinal epithelium is routinely renewed or rapidly repaired in the case of damage, a process driven by intestinal stem cells (ISCs) in mucosal glands known as crypts.^[^ ^1^, ^2^, ^3^ ^]^ Lgr5^+^ stem cells and crypt base columnar stem cells are intercalated with Paneth cells in the crypt, continuously generating rapidly proliferating transit‐amplifying cells that occupy the remainder of the crypt.^[^ ^4^ ^]^ Subsequently, transit‐amplifying cells differentiate into various intestinal epithelial cells (IECs), including enterocytes, tuft cells, goblet cells, and enteroendocrine cells.^[^ ^4^ ^]^

The intestinal epithelium is frequently vulnerable to malignant transformation and neoplasm. Colorectal cancer (CRC) is the third most common cancer worldwide, and is the fourth most common cause of cancer‐related death globally.^[^ ^5^ ^]^ Growing evidence revealed that only a group of cells within tumors, namely, cancer stem cells (CSCs), have full potential for tumorigenesis; these are the genuine drivers of tumor development.^[^ ^6^ ^]^ For example, Lgr5^+^ cells not only serve as stem cells for tissue renewal and regeneration in intestinal homeostasis, they can also fuel the pool of CSCs in intestinal tumorigenesis.^[^ ^7^, ^8^ ^]^ It is now understood that multiple signaling pathways control both ISCs and CSCs during this process, among which the Wnt/*β*‐catenin signaling has been extensively investigated over the past several decades.

The evolutionarily conserved Wnt pathway has been well documented for its essential role in individual development, tissue homeostasis, and tumorigenesis. In particular, over 90% of CRC patients show aberrant activation of this pathway with sustained accumulation of the downstream transcription coactivator *β*‐catenin in the nucleus.^[^ ^9^ ^]^ For example, deficiency of the tumor suppressor adenomatous polyposis coli (APC) protein, an upstream component of the Wnt pathway, acutely activates *β*‐catenin in ISCs, thusly promoting the development of CRC.^[^ ^10^, ^11^, ^12^, ^13^ ^]^ Depletion of *β*‐catenin has been shown to induce apoptosis and ablation of crypts in intestinal organoids.^[^ ^14^ ^]^ Yet currently the regulatory mechanisms of *β*‐catenin in ISC homeostasis and tumorigenesis are not fully understood.

In the absence of the Wnt signal, phosphorylation of *β*‐catenin at T45 by CK1*α* primes the subsequent phosphorylation of *β*‐catenin at S33/37/T41 by GSK3*β*, leading to *β*‐TrCP‐mediated degradation of *β*‐catenin.^[^ ^15^, ^16^, ^17^, ^18^, ^19^ ^]^ In contrast, when the Wnt signal is present, it interacts with Frizzled family receptors and LRP5/6 co‐receptors, and these interactions free *β*‐catenin from the APC‐containing destruction complex, generating a nonphosphorylated form of *β*‐catenin that can shuttle between the cytoplasm and nucleus. After entering the nucleus, *β*‐catenin binds to and transactivates the transcription factor TCF, which induces the expression of downstream target genes.^[^ ^19^, ^20^, ^21^, ^22^, ^23^, ^24^, ^25^, ^26^ ^]^ Beyond this classic scenario, a number of studies have hinted at additional mechanisms for the regulation of *β*‐catenin activity.^[^ ^27^, ^28^, ^29^, ^30^, ^31^, ^32^ ^]^ To date, however, such alternative molecular mechanism essential for full activation of *β*‐catenin remains partially understood and yet to be established in vivo. Moreover, these ideas about an expanded scope of *β*‐catenin biology have not been examined in ISCs or CSCs, leaving any possible role in tissue homeostasis and tumorigenesis unexplored.

The serine/threonine kinase MST4 belongs to the mammalian Ste20 family, members of which also include MST1/2 known as central kinases of the Hippo signaling pathway.^[^ ^33^, ^34^, ^35^ ^]^ Despite their structural similarities, the MST4 kinase is evolutionarily more distant to other members.^[^ ^36^ ^]^ MST4 has been implicated in biological processes including cell polarity, migration, autophagy, oxidative stress, and inflammation, as well as tumorigenesis.^[^ ^37^, ^38^, ^39^, ^40^, ^41^, ^42^, ^43^ ^]^ For example, MST4 can phosphorylate Ezrin and thereby inducing cell polarity.^[^ ^37^ ^]^ Also, MST4 can directly phosphorylate TRAF6 in macrophage to prevent excessive inflammation, therefore avoiding tissue damage.^[^ ^43^ ^]^ Recently, we discovered that MST4 acts as a noncanonical branch of the Hippo pathway to limit stress‐induced activation of the transcriptional coactivator YAP during gastric tumorigenesis.^[^ ^44^ ^]^

In this study, we characterized MST4 as a direct bona‐fide kinase of *β*‐catenin, which phosphorylates *β*‐catenin at Thr40 to inhibit GSK3*β*‐mediated phosphorylation at Ser33, therefore preventing its interaction with *β*‐TrCP. In response to Wnt signaling, this MST4‐p*β*‐catenin^Thr40^ axis is required for the process that actively blocks *β*‐TrCP‐mediated degradation, causing subsequent accumulation and full activation of *β*‐catenin. Disruption of the MST4‐p*β*‐catenin^Thr40^ axis in mice not only sharply decreased the number of ISCs/CSCs, but also suppressed CRC development; while constitutive activation of this axis by either forced activation of MST4 kinase or mutation of *β*‐catenin mimicking Thr40 phosphorylation led to increased number of ISCs/CSCs and exacerbated tumorigenesis. Consistently, the MST4‐p*β*‐catenin^Thr40^ signaling was markedly enhanced in CRC and associated with poor prognosis. Overall, this work uncovered a Wnt signaling‐induced MST4‐p*β*‐catenin^Thr40^ axis essential for ISC‐mediated tissue homeostasis and tumorigenesis, opening a window for novel targeted therapies.

## 2 Results

### 2.1 MST4 Is Essential for *β*‐Catenin Accumulation and Colorectal Cancer Cell Growth

**Figure 1:**
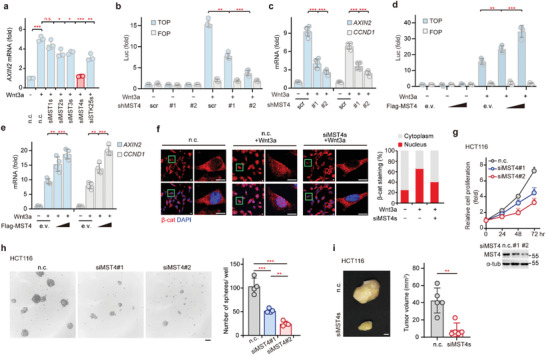
MST4 positively regulates Wnt signaling and colorectal cancer growth. a) Miniscreening for *AXIN2* transcription in Wnt3a‐stimulated HEK293FT cells transfected with siRNAs (two individual siRNAs/gene) targeting MST1, MST2, MST3, MST4, and STK25, respectively (*n* = 3). b) TOP‐FLASH reporter activity in MST4‐depleted HEK293FT cells following Wnt3a stimulation (*n* = 4). c) Expression of *AXIN2* and *CCND1* in MST4‐depleted cells after Wnt3a treatment (*n* = 6). d) TOP‐FLASH transactivity in MST4‐overexpressed cells after Wnt3a treatment (*n* = 4). e) Transcription of the indicated genes in MST4‐exprsessing cells after Wnt3a treatment (*n* = 4). f) Nuclear localization of *β*‐catenin in MST4‐knockdown cells following Wnt3a treatment. Scale bar, 10 µm. g) Cell proliferation of MST4‐depleted HCT116 cells at the indicated time course (*n* = 3). h) Sphere formation of HCT116 cells at day 10 after seeding into 6‐well ultralow attachment plates (*n* = 4). Scale bar, 100 µm. i) Tumor volume of HCT116 cells after being injected subcutaneously into nude mice for 14 days (*n* = 5). Scale bars, 1 mm. a,f–i) n.c., negative control. c) scr, scramble shRNA. b,c) shMST4, MST4 shRNA. d,e) e.v., empty vector. f,i) siMST4s, mixture of siMST4#1 and siMST4#2. a–h) Data represent the means ± SD. One‐way ANOVA with post hoc Bonferroni *t*‐test for multiple comparisons. i) Two‐tailed unpaired Student's *t*‐test for two variances, n.s., no significance; *p < 0.05; **p < 0.01; ***p < 0.001. See also Figure S1 in the Supporting Information.

Considering the multilayered crosstalk between the Hippo and Wnt pathways, we performed a bioinformatic analysis of the Hippo‐like MST family of kinases in a context of CRC, a type of cancer in which Wnt/*β*‐catenin is known to play a dominant role. We first analyzed transcription of MST kinases in CRC specimens from GEO Database (GSE 20842) and TCGA Database, and found in both cases that the expression levels of the MST4 kinase are significantly elevated in CRC samples compared with healthy tissues (*p* < 0.001; Figure S1a,b, Supporting Information). Moreover, the mRNA levels of *MST4* are positively correlated with those of *AXIN2*, a downstream target gene of Wnt pathway (Figure S1c, Supporting Information). To verify this phenomenon, we then knocked down each MST kinase in HEK293FT cells and performed QPCR assay on Wnt3a‐induced transcription of *AXIN2*. Intriguingly, we found knockdown of MST4 significantly decreased the mRNA levels of *AXIN2* (**Figure** **1**a; Figure S1d, Supporting Information). However, knockdown of MST1 did not affect *AXIN2* expression, whereas MST2/3 and STK25 only showed marginal effect (Figure 1a). Confirming MST4's regulatory effect on Wnt signaling, knockdown of MST4 strongly inhibited Wnt3a‐induced luciferase reporter activity of TOP but not of FOP (Figure 1b; Figure S1e, Supporting Information), and also significantly reduced the expression of *CCND1*, another Wnt target gene (Figure 1c). In contrast, overexpression of MST4 dose‐dependently enhanced Wnt3a‐induced TOP‐FLASH reporter activity, as well as the transcription of Wnt target genes (Figure 1d,e; Figure S1f, Supporting Information).

Since Wnt3a‐induced accumulation and subsequent nuclear translocation of *β*‐catenin are hallmarks of the Wnt signaling,^[^ ^20^, ^45^ ^]^ we next investigated the possible effect(s) of MST4 on *β*‐catenin. Consistent with MST4's positive regulation of Wnt signaling observed above, knockdown of MST4 in 3T3‐L1 cells substantially impaired Wnt3a‐induced nuclear translocation of *β*‐catenin (Figure 1f). Moreover, knockdown of MST4 not only impaired the proliferation of colon carcinoma cell line HCT116 (Figure 1g), but also inhibited HCT116‐derived sphere formation (Figure 1h). Furthermore, depletion of MST4 markedly suppressed tumor growth in HCT116‐derived xenograft model (Figure 1i), as well as in mice injected with MC38, a mouse colon carcinoma cell line (Figure S1g, Supporting Information).

Taken together, these lines of evidences strongly indicate an essential role of MST4 for Wnt/*β*‐catenin signaling and CRC growth.

### 2.2 MST4 Directly Interacts with and Phosphorylates *β*‐Catenin at Thr40

**Figure 2:**
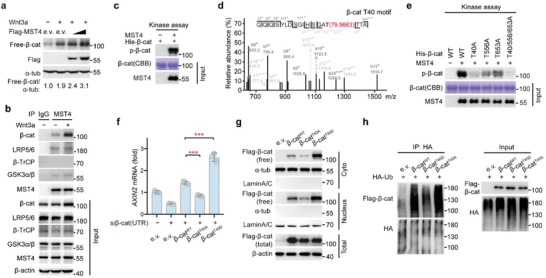
MST4 phosphorylates *β*‐catenin at Thr40 to block its ubiquitination and degradation by *β*‐TrCP. a) Immunoblotting of the Free‐*β*‐cat in MST4‐overexpressing cells after Wnt3a treatment for 2 h (*n* = 3). b) Co‐immunoprecipitation (Co‐IP) assay of the endogenous proteins to detect association of MST4 with *β*‐cat, LRP5/6, *β*‐TrCP, and GSK3*α*/*β* in response to Wnt3a treatment (*n* = 2). c) In vitro kinase assay of the *β*‐catenin by MST4. The phosphorylated proteins were detected using a thiophosphate‐ester‐specific antibody. CBB, Coomassie Brilliant Blue. d) Mass spectrometry analysis to detect *β*‐catenin phosphorylation at Thr40 by MST4. e) In vitro kinase assay to verify the phosphorylation sites of *β*‐catenin by MST4. CBB, Coomassie Brilliant Blue. f) *AXIN2* transcription in *β*‐cat‐depleted cells transfected with *β*‐catenin and its mutants. si*β*‐cat(UTR), *β*‐cat siRNAs targeting the UTR region of *β*‐cat (*n* = 3). Data represent means ± SD. One‐way ANOVA with post hoc Bonferroni *t*‐test for multiple comparisons, ***p < 0.001. g) Immunoblotting of the indicated proteins in cells after transfection with *β*‐catenin and its mutants. Cyto, cytoplasm (*n* = 2). h) Ubiquitination of *β*‐catenin and its mutants. a,f–h) e.v., empty vector. See also Figure S2 in the Supporting Information.

Cytoplasmic accumulation of *β*‐catenin protein is required for its translocation into nucleus.^[^ ^46^ ^]^ We performed western blotting assay and found depletion of MST4 in HEK293FT cells decreased the amount of Wnt3a‐induced free *β*‐catenin—a soluble form of *β*‐catenin^[^ ^28^, ^32^, ^47^ ^]^ (see details in the Experimental Section) (Figure S2a, Supporting Information). In contrast, overexpression of MST4 dose‐dependently increased the amount of free *β*‐catenin (**Figure** **2**a). Then, to evaluate whether the observed MST4‐mediated *β*‐catenin accumulation requires its kinase activity, we generated two variants of MST4 on its wildtype basis, namely an inactive K53R mutant (MST4^KR^), and a phosphomimic T178E mutant (MST4^TE^). We first depleted endogenous MST4 based on sgRNA targeting of the *MST4* UTR region in HEK293FT cells. As expected, MST4 deficiency abrogated Wnt3a‐induced transcription of *AXIN2* (Figure S2b,c, Supporting Information). Subsequent back transfection of wildtype MST4 but not the kinase‐dead MST4^KR^ variant rescued the Wnt3a‐induced *AXIN2* expression; while the constitutively active MST4^TE^ variant fully rescued the transcription of *AXIN2* (Figure S2b,c, Supporting Information). These observations clearly indicate that the kinase activity of MST4 is required for its regulatory effect on *β*‐catenin.

To explore the mechanism through which MST4 regulates *β*‐catenin accumulation, we next examined the possible physical association of MST4 with *β*‐catenin. Our co‐immunoprecipitation (co‐IP) assay in HEK293FT cells showed an interaction of endogenous MST4 with endogenous Wnt signaling key components LRP5/6, GSK3*α*/*β*, and *β*‐catenin but not E3 ligase *β*‐TrCP; note that Wnt3a stimulation especially enhanced this interaction of MST4 with *β*‐catenin (Figure 2b). To further dissect the MST4‐*β*‐catenin interaction, we purified proteins of maltose binding protein (MBP)‐tagged MST4 and His‐tagged *β*‐catenin and analyzed their interaction using a pull‐down approach, and our results indicate that MST4 directly interacts with *β*‐catenin (Figure S2d, Supporting Information).

Our findings that MST4 interacts with *β*‐catenin and promotes its accumulation in a kinase activity‐dependent manner prompted us to speculate that *β*‐catenin may be a substrate of MST4. To test this possibility, we performed an in vitro kinase assay using purified recombinant proteins of MST4 and *β*‐catenin, which revealed that *β*‐catenin was robustly phosphorylated when co‐incubated with MST4 (Figure 2c), thereby defining *β*‐catenin as a novel MST4 phosphorylation target. To determine the specific residue(s) of *β*‐catenin phosphorylated by MST4, we performed mass spectrometry‐based phosphoproteomics analysis, which identified several residues including Thr40, Thr556, and Thr653 as candidate sites (Figure 2d; Figure S2e,f, Supporting Information).

Next, we utilized a point mutation strategy to probe which residue of *β*‐catenin is the key phosphorylation site mediated by MST4. To this end, we mutated each possible phosphorylation site to alanine (A) and purified the corresponding *β*‐catenin proteins. Our in vitro kinase assay showed that mutation of Thr40 nearly blocked *β*‐catenin phosphorylation by MST4, while mutation of T556 and 653 reduced such phosphorylation (Figure 2e). Together, these results established that *β*‐catenin is a bona‐fide substrate of MST4 kinase, and indicate that Thr40 of *β*‐catenin is a major site targeted for phosphorylation by MST4.

### 2.3 Phospho‐Thr40 Causes *β*‐Catenin Accumulation via Blocking *β*‐TrCP‐Mediated Degradation

Given our finding that MST4 is essential for the accumulation of *β*‐catenin, we reasoned that the phosphorylation of *β*‐catenin's Thr40 residue may protect *β*‐catenin from degradation. To test this possibility, we mutated Thr40 to aspartate and anticipated that, if this *β*‐catenin^T40D^ mutant variant did mimic Thr40 constitutive phosphorylation as expected, then we would expect to find an increased *β*‐catenin accumulation, and increased transcription of downstream target genes. A rescue assay showed that cells expressing the *β*‐catenin^T40D^ variant had significantly increased transcription of the Wnt target gene *AXIN2* compared to cells with wildtype *β*‐catenin, but the transcription of *AXIN2* was significantly reduced in *β*‐catenin^T40A^ cells (Figure 2f). Consistent with these results, the fraction of free *β*‐catenin was apparently greater for the *β*‐catenin^T40D^ mutant than for wildtype *β*‐catenin (Figure 2g). In contrast, *β*‐catenin^T40A^, a mutant variant that cannot be phosphorylated at Thr40, showed a significantly decreased fraction of free *β*‐catenin (Figure 2g).

To further dissect the molecular mechanism linking *β*‐catenin Thr40 phosphorylation to its accumulation, we carried out immunoprecipitation coupled to mass spectrometry (IP‐MS) in HEK293FT cells that were transfected with wildtype *β*‐catenin or *β*‐catenin^T40A^ mutant. A total of 468 unique proteins were identified in our data analysis when using a protein false discovery rate equal to or lower than 1%. We then applied a manual thresholding approach and a probabilistic PPI prediction algorithm (SAINTexpress) to compute the most likely associations between each of these 468 proteins and *β*‐catenin (T40A). These methods yielded 40 high‐confidence candidates (Figure S2g, Supporting Information). These 40 proteins were at least twofold more abundant in the *β*‐catenin^T40A^ group than in the wildtype control, suggesting their specific associations with the *β*‐catenin^T40A^ variant. Functional enrichment analysis of the 40 *β*‐catenin^T40A^‐interacting proteins using the Kyoto Encyclopedia of Genes and Genomes (KEGG) database indicated that 25% of these proteins are cytoskeletal proteins and that 15% of them have annotated functions relating to protein degradation (Figure S2g, Supporting Information). We next performed IP experiments, which revealed that the *β*‐catenin^T40A^ variant was ubiquitinated to a significantly higher extent compared to wildtype *β*‐catenin; whereas the *β*‐catenin^T40D^ variant showed a decreased level of ubiquitination (Figure 2h). In keeping with this observation, the *β*‐catenin^T40D^ variant showed markedly reduced interaction with *β*‐TrCP, the E3 ubiquitin ligase of *β*‐catenin (Figure S2h, Supporting Information).

Overall, we concluded that MST4‐mediated Thr40 phosphorylation of *β*‐catenin blocks its binding to *β*‐TrCP and subsequent degradation, leading to accumulation of *β*‐catenin.

### 2.4 An MST4‐p*β*‐Catenin^Thr40^ Axis Actively Blocks GSK3*β* Phosphorylation of *β*‐Catenin at Ser33

**Figure 3:**
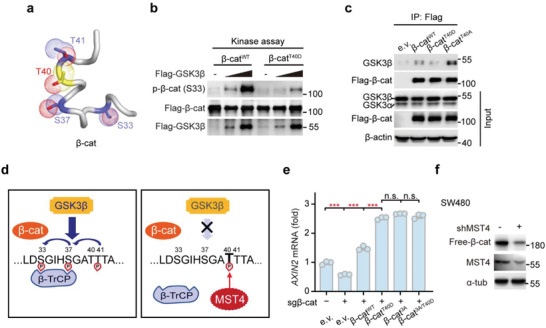
Thr40 phosphorylation inhibits GSK3*β* binding/phosphorylating *β*‐catenin. a) A structural model of phosphorylation sites of *β*‐catenin by GSK3*β* (Ser33/37/Thr41) and MST4 (Thr40). b) In vitro kinase assay of p‐*β*‐cat (S33) by GSK3*β*. The indicated proteins were purified using Flag beads (*n* = 2). c) Co‐IP assay to detect the interaction of GSK3*β* with *β*‐catenin and its mutants (*n* = 2). d) An illustration of p‐*β*‐cat (T40) by MST4 able to block the p‐*β*‐cat (S33) by GSK3*β*. e) mRNA levels of *AXIN2* in sg*β*‐cat‐treated cells after transfection with the indicated plasmids (*n* = 3). Data represent means ± SD. One‐way ANOVA with post hoc Bonferroni *t*‐test for multiple comparisons, ***p < 0.001; n.s., no significance. f) Immunoblotting of Free‐*β*‐cat in SW480 cells after infection with shMST4 lentivirus (*n* = 2). c,e) e.v., empty vector. See also Figure S3 in the Supporting Information.

It is known that GSK3*β* sequentially phosphorylates Thr41, Ser37, and then Ser33 residues in the N‐terminal of *β*‐catenin, and such phosphorylation facilitates *β*‐TrCP recognition, therefore serving as a degradation signal in the classic Wnt pathway.^[^ ^48^, ^49^ ^]^ Interestingly, our structural examination of *β*‐catenin indicated that the MST4‐targeting site Thr40 is in close vicinity to the GSK3*β*‐targeting cluster (**Figure** **3**a), hinting at possible interplay between the MST4‐p*β*‐catenin^Thr40^ axis and the classic Wnt signaling. To test this hypothesis, we performed in vitro kinase assays using immunoprecipitated Flag‐tagged *β*‐catenin^T40D^, *β*‐catenin^WT^, and GSK3*β*/CK1*α* proteins. Results showed that GSK3*β* dose‐dependently increased the phosphorylation level of wildtype *β*‐catenin at S33 site; while such effect was apparently reduced in the case of the *β*‐catenin^T40D^ variant (Figure 3b). Moreover, the *β*‐catenin^T40D^ variant showed a reduced interaction with GSK3*β*; while the *β*‐catenin^T40A^ variant showed an increased one (Figure 3c). These results indicate that the phosphorylation of *β*‐catenin at Thr40 site by MST4 competitively inhibits GSK3*β* binding and phosphorylating *β*‐catenin, which as a result prevents *β*‐TrCP‐mediated degradation of *β*‐catenin (Figure 3d).

To further assess the functional importance of the MST4‐p*β*‐catenin^Thr40^ axis in a context of classic Wnt signaling, we generated two *β*‐catenin mutants with one resistant to GSK3*β* phosphorylation (Ser33A/Ser37A/Thr41A, termed *β*‐catenin^3A^) and the other mimicking MST4 phosphorylation based on *β*‐catenin^3A^ (Ser33A/Ser37A/Thr41A/Thr40D, termed *β*‐catenin^3A/T40D^). We then obtained *β*‐catenin KO cells using CRISPR‐Cas9 method and performed a rescue assay in HEK293FT cells. Confirming the results of the in vitro kinase assay, we found wildtype *β*‐catenin rescued *AXIN2* transcription, and *β*‐catenin^T40D^ further increased *AXIN2* expression (Figure 3e; Figure S3a,b, Supporting Information). Importantly, *β*‐catenin^T40D^, *β*‐catenin^3A^, and *β*‐catenin^3A/T40D^ variants all promoted *AXIN2* expression to almost the same extent (Figure 3e; Figure S3a,b, Supporting Information), indicating that MST4 regulates the activity of *β*‐catenin mainly through blocking GSK3*β*‐mediated phosphorylation of *β*‐catenin.

Also, we explored the role(s) of MST4 on the activation of *β*‐catenin in SW480 cells, which harbor high levels of *β*‐catenin activity due to their APC deficiency.^[^ ^50^, ^51^, ^52^ ^]^ To our surprise, knockdown of MST4 in SW480 cells also reduced the levels of free *β*‐catenin (Figure 3f), as well as the TOP luciferase reporter activity and target gene transcription (Figure S3c,d, Supporting Information). Theoretically, APC mutation only partially activates Wnt signaling,^[^ ^53^, ^54^ ^]^ which may account for the currently observed effects of MST4 in SW480 cells. To test this possibility, we then fully activated Wnt signaling by LiCL stimulation. In this case, we found that knockdown or overexpression of MST4 did not significantly influence the transcription of *AXIN2* any more (Figure S3e,f, Supporting Information).

Taken together, these results demonstrate that in response to Wnt signaling, an MST4‐p*β*‐catenin^Thr40^ axis actively prevents the phosphorylation of *β*‐catenin by GSK3*β* and therefore blocks *β*‐TrCP‐mediated degradation, leading to the accumulation and full activation of *β*‐catenin.

### 2.5 Recapitulation of the MST4‐p*β*‐Catenin^Thr40^ Signaling Axis in the Intestinal Crypts

**Figure 4:**
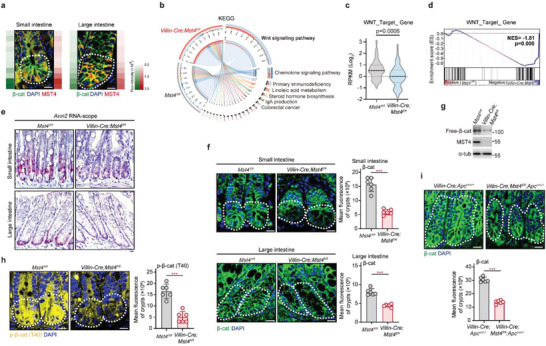
MST4 regulates *β*‐catenin accumulation in the intestinal crypts of mice. a) Representative micrographs of MST4 and *β*‐catenin in small intestines and large intestines from the wildtype mice. b) KEGG pathway enrichment analysis for RNA‐seq in primary intestinal epithelial cells from *Mst4^fl/fl^* and *Villin‐Cre;Mst4^fl/fl^* mice (*n* = 3). c) Violin plots showing the RPKM values of Wnt target genes in RNA‐seq data. d) GSEA analysis showing negative enrichment of Wnt target genes in the MST4‐depletion group. e) In situ hybridization of *Axin2* transcription in crypts of small intestines and large intestines (*n* = 6). f) Immunofluorescent staining of *β*‐catenin in intestinal crypts of *Mst4^fl/fl^* and *Villin‐Cre;Mst4^fl/fl^* mice (*n* = 6). g) Immunoblotting of Free‐*β*‐cat in intestinal epithelial cells from *Mst4^fl/fl^* and *Villin‐Cre;Mst4^fl/fl^* mice. h) Fluorescent staining to detect p‐*β*‐cat (T40) in the small intestines from *Mst4^fl/fl^* and *Villin‐Cre;Mst4^fl/fl^* mice (*n* = 6). i) Immunofluorescent analysis of *β*‐catenin in the small intestines from the indicated mice. a,e,f,h,i) Scale bar, 10 µm. c,f,h,i) Data represent means ± SD. Two‐tailed unpaired Student's *t*‐test for two variances. ***p < 0.001. See also Figure S4 in the Supporting Information.

After establishing in vitro the MST4‐p*β*‐catenin^Thr40^ singling axis, we carried on to investigate this axis in vivo. Given the realized importance of the classic Wnt signaling in intestinal tissue homeostasis and tumorigenesis,^[^ ^15^, ^55^, ^56^, ^57^, ^58^, ^59^, ^60^ ^]^ we first detected the expression of MST4 and *β*‐catenin in intestine and found both MST4 and *β*‐catenin were highly expressed at the bottom of small and large intestinal crypts (**Figure** **4**a). Consistent with our in vitro pulldown result showing a direct interaction between MST4 and *β*‐catenin, these two proteins seemingly colocalize with each other in the crypts (Figure 4a). Then, we crossed *Villin‐Cre* and *Mst4^flox/flox^* mice to create conditional knockout mice and silenced the expression of MST4 in intestinal epithelial cells (referred to as *Villin‐Cre;Mst4^fl/fl^*). Initial observations successfully experimentally confirmed the expected recombinant alleles of *Villin‐Cre;Mst4^fl/fl^* mice and absence of the MST4 protein after its *Villin*‐Cre‐mediated excision in IECs (Figure S4a,b, Supporting Information).

We next performed RNA sequencing analyses of IECs isolated from *Villin‐Cre;Mst4^fl/fl^* and wildtype mice. A KEGG pathway enrichment analysis on this RNA sequence data set suggested the involvement of MST4 in multiple biological processes, including signal transduction and colorectal cancer, and also in the Wnt signaling pathway (Figure 4b). Overall, the expression of Wnt target genes tended to be downregulated in IECs upon depletion of MST4 (Figure 4c). Subsequent gene set enrichment analysis (GSEA) revealed that knockout of MST4 in IECs significantly reduced the expression of many Wnt target genes (Figure 4d). Further qPCR analysis showed that the mRNA expression levels of Wnt target genes, including *Axin2*, *Ccnd1*, *Lgr5*, and *Sox9* were significantly decreased in *Villin‐Cre;Mst4^fl/fl^* mice (Figure S4c, Supporting Information). Subsequently, RNA scope results indicated MST4‐deficiency in IECs indeed markedly inhibited transcription of Wnt target gene *Axin2* (Figure 4e), confirming a positive regulatory role for MST4 in Wnt/*β*‐catenin signaling in the intestine.

As previously observed, MST4 promotes accumulation of *β*‐catenin by directly interacting with and phosphorylating *β*‐catenin (Figure 2). This was further confirmed by immunostaining showing that the *β*‐catenin protein levels were markedly reduced (≈66%) in the small intestinal crypt cells of *Villin‐Cre;Mst4^fl/fl^* mice as compared to control littermates, so were large intestinal crypts (Figure 4f). Consistent with these observations, the amounts of free *β*‐catenin were substantially decreased in MST4‐depleted IECs (Figure 4g).

We then generated an antibody that specifically recognizes *β*‐catenin phosphorylated at Thr40. To verify this function of the antibody, we coexpressed Flag‐tagged *β*‐catenin and Myc‐tagged MST4 in HEK293FT cells. The Thr40‐phospho‐specific antibody readily detected the phosphorylation of wildtype *β*‐catenin but not the *β*‐catenin^T40A^ variant (Figure S4d, Supporting Information). Besides, wildtype MST4 but not the kinase‐dead MST4^KR^ variant was able to increase phosphorylation of *β*‐catenin at Thr40, with the MST4^TE^ variant causing yet further increases in *β*‐catenin phosphorylation at Thr40 (Figure S4e, Supporting Information), confirming the specificity of the Thr40‐phospho antibody and Thr40 as a primary phosphorylation site targeted by MST4. By using this Thr40‐phospho‐specific antibody, we found the levels of endogenous p*β*‐catenin^Thr40^ is sharply increased in response to Wnt3a stimulation (Figure S4f,g, Supporting Information). We then used this antibody to examine the levels of p*β*‐catenin^Thr40^ in the mouse intestine. The pattern of fluorescence staining here was roughly consistent with that of total *β*‐catenin staining in wildtype IECs (Figure 4f), with the strongest signal in crypt cells: the level of p*β*‐catenin^Thr40^ staining in *Villin‐Cre;Mst4^fl/fl^* mice was much lower than that in control littermates (Figure 4h).

Also, we assessed the regulatory effect of MST4 in *Apc^min/+^* mice, which have been shown to accumulate *β*‐catenin.^[^ ^61^ ^]^ The protein levels of *β*‐catenin were apparently decreased in the intestinal crypts of *Villin‐Cre;Mst4^fl/fl^;Apc* ^min/+^ mice compared to *Villin‐Cre;Apc^min/+^* mice (Figure 4i), findings consistent with our observations in SW480 cells (Figure 3f) showing that MST4 is required for the accumulation of *β*‐catenin even in cells with partially activated Wnt signaling.

Taken together, these results recapitulate the MST4‐p*β*‐catenin^Thr40^ signaling axis in the intestinal crypts.

### 2.6 Disruption of the MST4‐p*β*‐Catenin^Thr40^ Axis Results in Loss of ISC/CSCs and Inhibition of CRC

**Figure 5:**
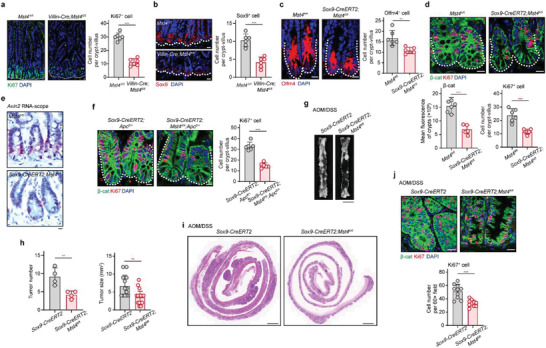
Deficiency of MST4 causes loss of ISCs and inhibits ISC‐driven CRC development. a,b) Immunofluorescent staining of Ki67 and Sox9 in murine small intestines from *Mst4^fl/fl^* and *Villin‐Cre;Mst4^fl/fl^* mice (*n* = 6). Ki67, proliferating cell marker. Sox9, stem cell marker. c) Fluorescent staining of Olfm4^+^ cells in the intestinal crypts from *Mst4^fl/fl^* and *Sox9‐CreERT2;Mst4^fl/fl^* mice (*n* = 6). d,f) Immunofluorescence of *β*‐catenin and Ki67 in crypts of the indicated mice (*n* = 6). e) In situ hybridization of *Axin2* in small intestines and large intestines (*n* = 6). g) Image of large intestinal tumors from *Sox9‐CreERT2* and *Sox9‐CreERT2;Mst4^fl/fl^* mice after AOM/DSS challenge (*n* = 4). Scale bars, 10 mm. h) Number and size of tumors in the AOM/DSS‐treated mice (*n* = 4). i) H&E staining of tumor tissues from *Sox9‐CreERT2* and *Sox9‐CreERT2;Mst4^fl/fl^* mice (*n* = 4). Scale bars, 1 mm. j) Immunofluorescent staining of *β*‐catenin and Ki67 in tumors from *Sox9‐CreERT2* and *Sox9‐CreERT2;Mst4^fl/fl^* mice (*n* = 4). a–f,j) Scale bar, 10 µm. a–d,f,h,j) Data represent means ± SD. Two‐tailed unpaired Student's *t*‐test for two variances, **p < 0.01; ***p < 0.001. See also Figure S5 in the Supporting Information.

Intestinal stem cells at the bottom of crypts can proliferate and differentiate into various types of functional epithelial cells including Goblet cells and Paneth cells.^[^ ^62^, ^63^ ^]^ To examine the potential physiological functions of MST4‐p*β*‐catenin^Thr40^ axis in intestinal homeostasis, we examined the proliferation of epithelial cells in *Villin‐Cre;Mst4^fl/fl^* mice. Histological analysis with Ki67 immunostaining indicated an intense proliferative state of crypt cells in wildtype mice, while such proliferation was apparently reduced in the *Villin‐Cre;Mst4^fl/fl^* mice (**Figure** **5**a). Consistent with this observation, markedly fewer Sox9^+^ and Olfm4^+^ ISCs were observed in the crypts of *Villin‐Cre;Mst4^fl/fl^* mice than those in wildtype mice (Figure 5b; Figure S5a, Supporting Information). It is conceivable that a decreased number of ISCs would affect Paneth and Goblet cells. Indeed, quantification of these cells revealed that *Villin‐Cre;Mst4^fl/fl^* mice had much fewer Paneth cells than control mice (Figure S5b, Supporting Information), as well as a decreased number of Goblet cells (Figure S5c, Supporting Information). As a result, the small intestines of the *Villin‐Cre;Mst4^fl/fl^* mice were significantly shorter than those of the control littermates (Figure S5d, Supporting Information).

Given that ISCs are required for intestinal tissue regeneration, we reasoned that deletion of MST4 would block this process due to loss of ISCs. To test this hypothesis, we crossed *Mst4‐floxed* mice with *Lgr5‐EGFP‐ires‐CreERT2* mice to generate tamoxifen‐inducible ISC‐specific knockout mice (referred to as *Lgr5‐CreERT2;Mst4^fl/fl^*). First, we selectively killed proliferating cells in intestinal epithelium using 5‐fluoruracil (5‐FU) as reported previously.^[^ ^64^ ^]^ Staining for Ki67 confirmed that proliferating cells completely disappeared after 2 days post intraperitoneal injection of 5‐FU (Figure S5e, Supporting Information). At day 3, Ki67^+^ cells were rapidly increased to repair the 5‐FU‐induced damage in control mice, whereas these cells were rarely present in *Lgr5‐CreERT2;Mst4^fl/fl^* mice (Figure S5f, Supporting Information). Furthermore, a contribution of Lgr5^+^ cells to regeneration postinduction was seen after 3 days and gradually expanded throughout crypt‐villus in control mice. However, Lgr5^+^ ISCs were hardly observed in *Lgr5‐CreERT2;Mst4^fl/fl^* mice, and the regeneration was blocked in these mice (Figure S5g, Supporting Information).

Next, we crossed *Mst4‐floxed* mice with *Sox9‐ires‐CreERT2* mice to generate tamoxifen‐inducible ISC‐specific knockout mice (referred to as *Sox9‐CreERT2;Mst4^fl/fl^*). Consistent with the observations in *Villin‐Cre;Mst4^fl/fl^* mice, MST4 deficiency in Sox9^+^ cells markedly weakened the number of stem cells (Olfm4^+^ cells) and proliferating cells (Ki67^+^ cells) (Figure 5c,d). Besides, staining results indicated that protein levels of *β*‐catenin were obviously decreased in crypts of *Sox9‐CreERT2;Mst4^fl/fl^* mice than that of control mice (Figure 5d). Moreover, RNA scope results revealed the transcription of Wnt/*β*‐catenin signaling target gene *Axin2* was sharply downregulated in *Sox9‐CreERT2;Mst4^fl/fl^* mice (Figure 5e), findings that support an essential role of the MST4‐p*β*‐catenin^Thr40^ axis in ISC homeostasis. Notably, the accumulation of *β*‐catenin and the number of proliferating cells were also significantly reduced in *Sox9‐CreERT2;Mst4^fl/fl^;Apc^fl/+^* mice compared with *Sox9‐CreERT2;Apc^fl/+^* mice (Figure 5f).

Finally, to investigate potential function(s) of the MST4‐p*β*‐catenin^Thr40^ axis in CRC, we first examined CRC tissue samples from mice treated with azoxymethane/dextran sodium sulfate (AOM/DSS). As shown in Figure 5g, these mice developed colorectal adenocarcinoma at about 3 months with 4 cycles of AOM/DSS induction. Importantly, specific depletion of MST4 in ISCs (*Sox9‐CreERT2;Mst4^fl/fl^*) significantly decreased colorectal tumorigenesis (Figure 5g,h). H&E staining also showed that depletion of MST4 in Sox9^+^ cells seemingly ameliorated colorectal carcinogenesis (Figure 5i). Meanwhile, the number of Ki67^+^ cells was evidently reduced in *Sox9‐CreERT2;Mst4^fl/fl^* mice compared with control mice during AOM/DSS‐induced CRC (Figure 5j).

Taken together, these results clearly demonstrate an essential role for the MST4‐p*β*‐catenin^Thr40^ axis in ISCs/CSCs homeostasis and CRC development.

### 2.7 Constitutive Activation of MST4 Kinase Increases the Number of ISCs/CSCs and Exacerbates CRC

**Figure 6:**
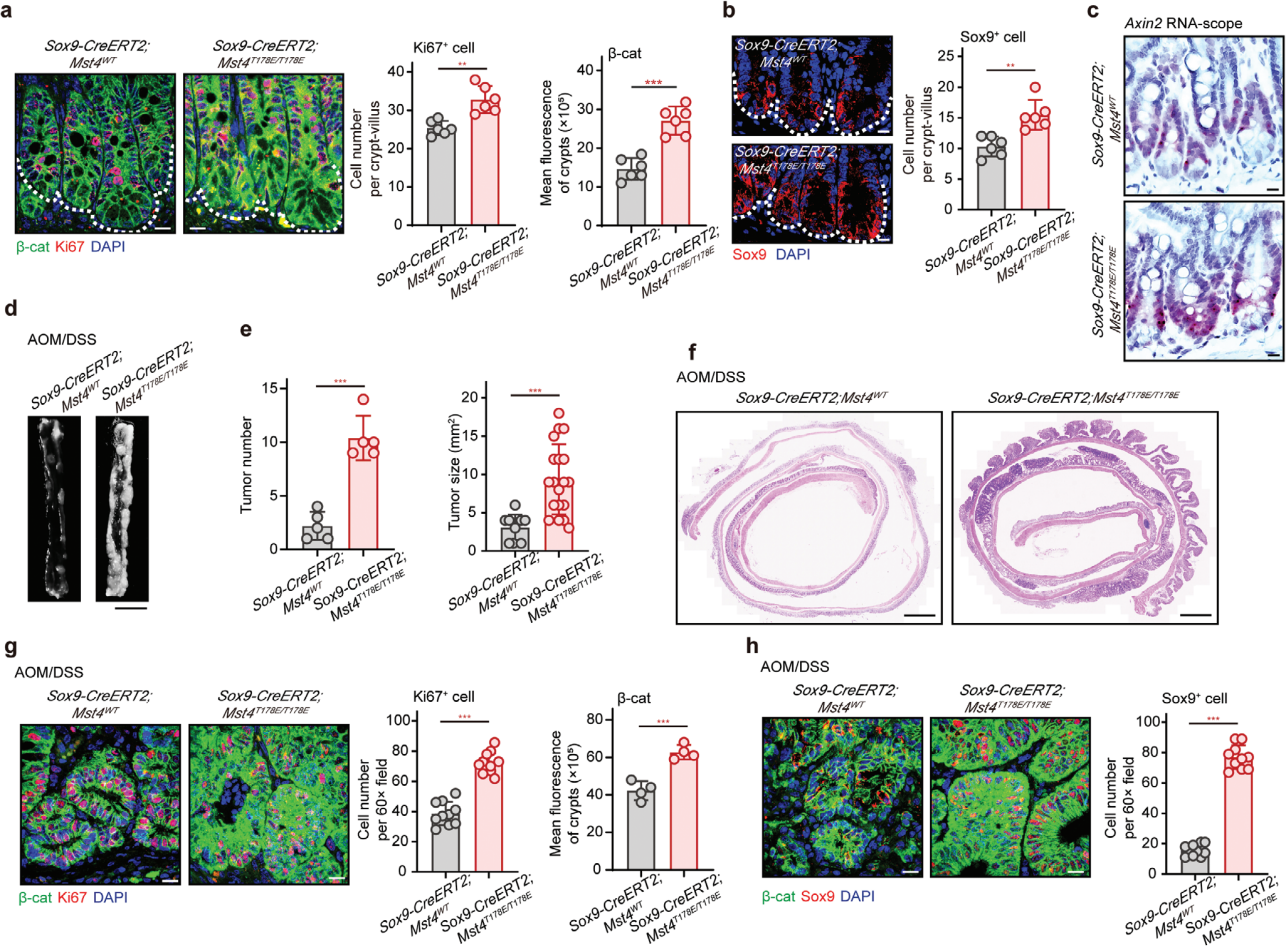
T178E mutation of MST4 increases ISC number and exacerbates ISC‐driven CRC. a) Immunofluorescence of *β*‐catenin and Ki67 in the small intestinal crypts from *Sox9‐CreERT2;Mst4^WT^* and *Sox9‐CreERT2;Mst4^T178E/T178E^* mice (*n* = 6). b) Fluorescent staining of Sox9^+^ cells in the intestinal tissues from the indicated mice (*n* = 6). c) In situ hybridization of *Axin2* transcription in the crypts of *Sox9‐CreERT2;Mst4^WT^* and *Sox9‐CreERT2;Mst4^T178E/T178E^* mice (*n* = 6). d) Images of tumor tissues from AOM/DSS‐challenged *Sox9‐CreERT2;Mst4^WT^* and *Sox9‐CreERT2;Mst4^T178E/T178E^* mice (*n* = 5). Scale bars, 10 mm. e) Number and size of tumors in the AOM/DSS‐treated mice (*n* = 5). f) H&E staining of tumor tissues from *Sox9‐CreERT2;Mst4^WT^* and *Sox9‐CreERT2;Mst4^T178E/T178E^* mice (*n* = 5). Scale bars, 1 mm. g) Immunofluorescence of *β*‐catenin and Ki67 in tumor tissues from *Sox9‐CreERT2;Mst4^WT^* and *Sox9‐CreERT2;Mst4^T178E/T178E^* mice (*n* = 5). h) Immunofluorescence of *β*‐catenin and Sox9 in tumor tissues from the indicated mice (*n* = 5). a–c,g,h) Scale bar, 10 µm. a,b,e,g,h) Data represent means ± SD. Two‐tailed unpaired Student's *t*‐test for two variances, **p < 0.01; ***p < 0.001. See also Figure S6 in the Supporting Information.

To examine the functional consequence upon hyperactivation of the MST4‐p*β*‐catenin^Thr40^ axis, we created *Mst4^T178E^* conditional knockin mice and crossed them with *Sox9‐CreERT2* mice (referred to as *Sox9‐CreERT2;Mst4^T178E/T178E^*) to endorse constitutive kinase activity of the MST4 in ISCs (Figure S6a,b, Supporting Information). In light of our mechanistic study, we reasoned that forced constitutive activation of MST4 would, by increasing phosphorylation at Thr40, stabilize and accumulate *β*‐catenin. Indeed, the protein levels of *β*‐catenin were significantly increased, with ≈50% higher abundance in crypt cells of the *Sox9‐CreERT2;Mst4^T178E/T178E^* mice than that in control littermates (**Figure** **6**a). A subsequent histochemistry analysis revealed Ki67^+^ proliferating cells and Sox9^+^ ISCs were evidently increased in the crypts of the *Sox9‐CreERT2;Mst4^T178E/T178E^* mice compared to control mice (Figure 6a,b). Furthermore, constitutive activation of MST4 in Sox9^+^ cells sharply promoted transcription of *β*‐catenin target gene *Axin2* (Figure 6c). In short, constitutive activation of MST4 in intestinal stem cells led to accumulation of *β*‐catenin and increased number of ISCs.

We next assessed the pathological consequence upon forced constitutive activation of MST4 in ISCs/CSCs (*SOX9‐CreERT2*) using the CRC mice model. Results showed that constitutive activation of MST4 in ISCs/CSCs significantly increased AOM/DSS‐induced colorectal tumorigenesis (Figure 6d,e). H&E results also found that MST4 activation in ISCs/CSCs evidently aggravated AOM/DSS‐induced CRC progression (Figure 6f). Moreover, accumulation of *β*‐catenin and Ki67^+^ cells were markedly increased in tumors of the *Sox9‐CreERT2;Mst4^T178E/T178E^* mice compared to control mice during CRC progression (Figure 6g). Consistently with these observations, constitutive activation of MST4 resulted in increased number of CSCs as shown by staining of Sox9 in CRC mice (Figure 6h).

Taken together, these results indicate that hyperactivation of the MST4‐p*β*‐catenin^Thr40^ axis increases the number of ISCs/CSCs and promotes colorectal tumorigenesis.

### 2.8 *Ctnnb1^T40D^* Mice Phenocopies *Mst4^T178E^* Mice Regarding ISCs/CSCs Number and CRC Development

**Figure 7:**
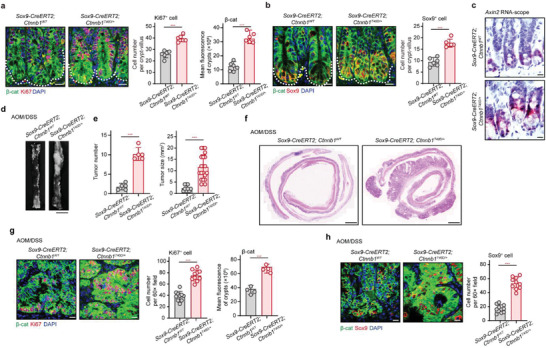
T40D mutation of *β*‐catenin increases ISC number and exacerbates ISC‐driven CRC. a) Immunofluorescent staining of *β*‐catenin and Ki67 in the small intestines of *Sox9‐CreERT2;Ctnnb1^WT^* and *Sox9‐CreERT2;Ctnnb1^T40D/+^* mice (*n* = 6). b) Immunofluorescence of *β*‐catenin and Sox9 in the small intestine of the indicated mice (*n* = 6). c) In situ hybridization of *Axin2* in the crypts of *Sox9‐CreERT2;Ctnnb1^WT^* and *Sox9‐CreERT2;Ctnnb1^T40D/+^* mice (*n* = 6). d) Gross image of colorectal tumors in *Sox9‐CreERT2;Ctnnb1^WT^* and *Sox9‐CreERT2;Ctnnb1^T40D/+^* mice after AOM/DSS treatment (*n* = 5). Scale bars, 10 mm. e) Number and size of tumors in the indicated mice (*n* = 5). f) H&E staining of AOM/DSS‐induced tumors from the *Sox9‐CreERT2;Ctnnb1^WT^* and *Sox9‐CreERT2;Ctnnb1^T40D/+^* mice (*n* = 5). Scale bars, 1 mm. g,h) Representative images of the indicated proteins in tumor tissues from *Sox9‐CreERT2;Ctnnb1^WT^* and *Sox9‐CreERT2;Ctnnb1^T40D/+^* mice (*n* = 5). a–c,g,h) Scale bar, 10 µm. a,b,e,g,h) Data represent means ± SD. Two‐tailed unpaired Student's *t*‐test for two variances, ***p < 0.001.

Based on our in vitro and in vivo dissection of the MST4‐p*β*‐catenin^Thr40^ axis, we reasoned that a *β*‐catenin mutant T40D (*Ctnnb1^T40D^*) mimicking constitutive phosphorylation of *β*‐catenin by MST4 would cause functional and/or pathological consequences similar to those observed for constitutive activation of MST4. To test this point, we created *Ctnnb1^T40D^* conditional knockin mice and crossed them with *Sox9‐CreERT2* mice (referred to as *Sox9‐CreERT2;Ctnnb1^T40D/+^*) to specifically mimic phosphorylation of *β*‐catenin by MST4 in ISCs. Indeed, we observed more accumulation of *β*‐catenin and stronger signal for Ki67 staining in crypt cells of the *Sox9‐CreERT2;Ctnnb1^T40D/+^* mice than in wildtype control mice (**Figure** **7**a). Consistent with these results, the number of Sox9^+^ ISCs was obviously increased in the crypts of the *Sox9‐CreERT2;Ctnnb1^T40D/+^* mice than in control littermates (Figure 7b). Moreover, RNA scope assay for the target gene *Axin2* further confirmed the elevated activity of *β*‐catenin once upon phosphor‐mimic mutation of Thr40 (Figure 7c).

Subsequently, we assessed the potential effect of *β*‐catenin T40D mutation in ISCs during AOM/DSS‐induced colorectal tumorigenesis. Similar to our observations in the *Sox9‐CreERT2;Mst4^T178E/T178E^* mice, AOM/DSS‐induced colorectal tumorigenesis was seemingly accelerated in the *Sox9‐CreERT2;Ctnnb1^T40D/+^* mice than in control littermates (Figure 7d,e). Further H&E staining confirmed the deteriorated tumorigenesis upon phosphor‐mimic mutation of *β*‐catenin Thr40 (Figure 7f). Moreover, the protein levels and nuclear localization of *β*‐catenin, as well as the number of proliferating cells were observably increased in tumors of the *Sox9‐CreERT2;Ctnnb1^T40D/+^* mice compared with control mice (Figure 7g). Importantly, the number of Sox9^+^ CSCs was also markedly increased in tumors of the *Sox9‐CreERT2;Ctnnb1^T40D/+^* mice compared with control mice (Figure 7h).

Together with the observations for MST4 knockout/knockin mice, these results demonstrated that hyperactivation of the MST4‐p*β*‐catenin^Thr40^ axis increases the number of Sox9^+^ stem cells and accelerates CRC progression.

### 2.9 The MST4‐p*β*‐Catenin^Thr40^ Axis Is Hyperactivated in CRC and Associated with Poor Prognosis

**Figure 8:**
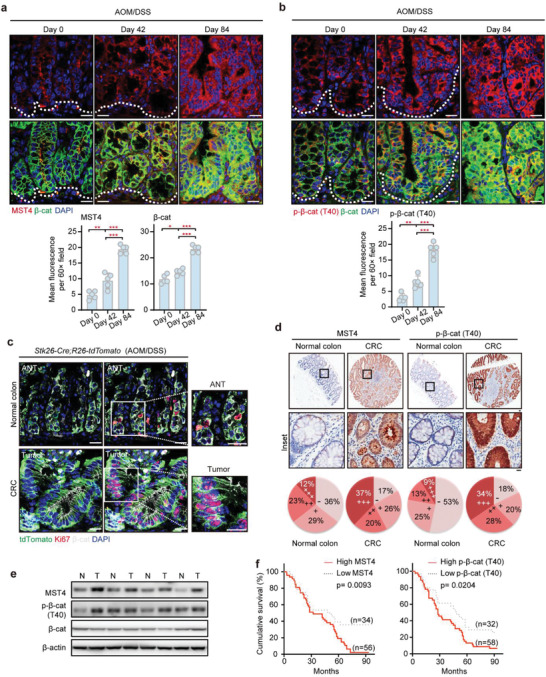
The MST4‐p*β*‐catenin^Thr40^ axis was hyperactivated in CRC and associated with worse prognosis. a,b) Immunofluorescent analysis of MST4/p‐*β*‐cat(T40) and *β*‐cat in AOM/DSS‐induced tumors. One‐way ANOVA with post hoc Bonferroni *t*‐test for multiple comparisons, *p < 0.05; **p < 0.01; ***p < 0.001. c) Representative micrographs of the indicated proteins in tumor and adjacent colon tissues from AOM/DSS‐treated *Stk26‐Cre;R26‐tdTomato* mice. d) Immunohistological staining of MST4 and p‐*β*‐cat(T40) in human CRCs and paired adjacent colon tissues. Scale bar, 20 µm. e) Immunoblotting of MST4 and p‐*β*‐cat(T40) in CRC specimens. f) Kaplan–Meier survival curves of patients on the expression of MST4 (left) and p‐*β*‐cat(T40) (right). Survival analysis was performed using a log‐rank test. a–c) Scale bar, 10 µm.

Given the above findings of the MST4‐p*β*‐catenin^Thr40^ axis in ISCs/CSCs homeostasis and CRC development, we speculated that this axis is hyperactivated during colorectal tumorigenesis and associated with bad clinical outcomes. To test this possibility, we first analyzed protein levels of MST4 and phosphorylation levels of *β*‐catenin Thr40 in AOM/DSS‐induced mouse CRC. Indeed, we observed a clear and progressive increase for the expression of both MST4 and p*β*‐catenin^Thr40^ along the process of CRC development (**Figure** **8**a,b). Subsequently, we created *Stk26‐Cre* mice (*Stk26* is the gene name of MST4) and crossed them with tdTomato mice (referred to as *Stk2* *6‐Cre*;*R26‐tdTomato*) to genetically trace cells expressing MST4 during AOM/DSS‐induced colorectal tumorigenesis. The results of tdTomato lineage tracing showed that MST4^+^ cells were mainly found at the bottom of the crypts for normal part of mouse colon (Figure 8c). However in the tumor tissue, MST4^+^ cells were markedly increased spreading all over the tumor tissue with significantly elevated levels of *β*‐catenin; staining signals for Ki67 were readily detected in these cells (Figure 8c).

Next, we examined the activation status of the MST4‐p*β*‐catenin^Thr40^ signaling axis in clinical samples of CRC patients. Consistent with the observations in CRC mice model, immunohistochemical (IHC) staining of tissue microarrays showed that MST4 protein levels were sharply increased in tumor tissues of CRC patients when compared with that of healthy control (Figure 8d). Correlated with the upregulated expression of MST4, the staining signal for our *β*‐catenin Thr40‐phosphorylation‐specific antibody was also significantly increased in CRC tissues as compared to normal tissues (Figure 8d). Moreover, western blotting of the CRC specimens revealed that elevated levels of MST4 protein were directly associated with the accumulation of the Thr40‐phosphorylated form of *β*‐catenin (Figure 8e).

Furthermore, we analyzed the potential correlation of MST4 expression levels with tumor size and tumor stage in 90 CRC patients (Table S1, Supporting Information). We found the expression levels of MST4 were positively correlated with tumor size (*p* = 0.0158), tumor stage (*p* = 0.0205), and lymph node metastasis (*p* = 0.0365). Also, the levels of *β*‐catenin Thr40 phosphorylation were positively correlated with tumor size (*p* = 0.046), tumor stage (*p* = 0.029), and lymph node metastasis (*p* = 0.035) (Table S2, Supporting Information). Meanwhile, the expression levels of MST4 were positively correlated with the levels of *β*‐catenin Thr40 phosphorylation (*p* = 0.011) (Table S3, Supporting Information). In addition, Kaplan–Meier survival analysis of the tissue microarray data revealed that expression levels of MST4 (*p* = 0.0093) and *β*‐catenin Thr40 phosphorylation levels (*p* = 0.0204) were both negatively correlated with the 7.5‐year survival rate for CRC patients (Figure 8f).

Taken together, these results indicate that the MST4‐p*β*‐catenin^Thr40^ axis is overly activated in CRC and such hyperactivation is correlated with worse clinical outcomes of CRC patients.

## 3 Discussion

Despite the central roles of Wnt signaling in cell stemness, tissue homeostasis, and tumorigenesis,^[^ ^59^, ^65^, ^66^ ^]^ therapeutic targeting of *β*‐catenin remains problematic due to incomplete understanding of the regulatory mechanism. Here, we uncovered a Wnt signal‐induced MST4‐p*β*‐catenin^Thr40^ axis essential for *β*‐catenin accumulation and ISC maintenance, and further demonstrated that this axis is hyperactivated in CRC and associated with poor prognosis (Figure S7, Supporting Information).

The classic Wnt signaling triggers the release of *β*‐catenin from the APC‐containing destruction complex, which prevents its phosphorylation by CK1*α*/GSK3*β* and subsequent *β*‐TrCP‐mediated ubiquitination and degradation.^[^ ^67^, ^68^ ^]^ However, it remains elusive whether additional mechanism is required to stop this GSK3*β*‐*β*‐TrCP‐mediated molecular process in response to Wnt signaling. Previous studies showed that Rac1/PAK1 phosphorylates *β*‐catenin to activate Wnt signaling.^[^ ^31^, ^32^ ^]^ In the current work, we found that an MST4‐p*β*‐catenin^Thr40^ signaling axis can respond to Wnt3a stimulation to actively block the GSK3*β*‐*β*‐TrCP signaling cascade, therefore leading to the accumulation and full activation of *β*‐catenin. Note that such effect was also observed in the case of partially activated Wnt signaling (SW480 cells, *Apc^min^* mice). Mechanistically, MST4 phosphorylates *β*‐catenin at Thr40 to competitively inhibit GSK3*β* binding/phosphorylating *β*‐catenin, preventing *β*‐TrCP from binding and degrading *β*‐catenin.

Our finding of the MST4‐p*β*‐catenin^Thr40^ axis confirms the importance of restricting *β*‐TrCP activity toward *β*‐catenin, and further highlights multiple‐layered regulations at this step. That said, we also observed, using IP‐MS, that the *β*‐catenin^T40A^ variant underwent increased associations with cytoskeleton‐related proteins compared to wildtype *β*‐catenin. At this stage, we cannot rule out the possibility of MST4 regulating *β*‐catenin via some adhesion‐based mechanism (for example, by directing its attachment to the cytoskeleton and/or membrane system). Further study will be needed to fully address the MST4‐mediated stabilizing mechanism of *β*‐catenin, especially in a context of multiple substrates (e.g., Ezrin, TRAF6, YAP) intertwined with each other. Nonetheless, our findings that the MST4‐p*β*‐catenin^Thr40^ axis is required for *β*‐catenin accumulation support the usage of Thr40 phosphorylation as a new marker for Wnt/*β*‐catenin signaling.

Supporting a protumorigenic role of the MST4‐p*β*‐catenin^Thr40^ axis, we found that MST4 was hyperactivated in both mouse and human CRC tissues, and that MST4 expression levels were positively correlated with CRC progression but negatively correlated with CRC patient survival. Importantly, the levels of phosphorylation of Thr40 of *β*‐catenin were dramatically increased, as detected by using a fully verified homemade Thr40‐phosphor‐specific antibody in CRC patients, indicative of hyperactivation of the MST4‐p*β*‐catenin^Thr40^ signaling in these patients. In this regard, whether the levels of Thr40 phosphorylation of *β*‐catenin could be considered as a diagnostic marker for CRC warrants further investigation using a larger sample size, and perhaps different subtypes of CRC.

Consistent with the importance of *β*‐catenin activity in stem cell biology, our work established an essential role of the MST4‐p*β*‐catenin^Thr40^ axis in maintaining ISCs/CSCs homeostasis in contexts of both physiology and tumorigenesis. Yet whether and how this axis governs other types of stem cells warrants further investigation. As aberrant activation of the *β*‐catenin in ISCs promotes adenomatous growth,^[^ ^7^, ^69^, ^70^ ^]^ it is likely that the MST4‐p*β*‐catenin^Thr40^ axis in ISCs became hyperactivated during tumorigenesis, and then directly promote the expansion of CSCs during CRC development. Supporting this notion, human and mouse CSCs indeed have severe upregulation of the MST4‐p*β*‐catenin^Thr40^ axis. More importantly, multiple means of genetic manipulation of this axis specifically in ISCs markedly altered the progression of CRC in mice models. For example, constitutive activation of MST4 (*Sox9‐CreERT2;Mst4^T178E/T178E^*) or heterozygous mutation of *β*‐catenin T40D mimicking its phosphorylation by MST4 in ISCs substantially increased the number of ISCs/CSCs and exacerbated CRC. Thus, hyperactivation of the MST4‐p*β*‐catenin^Thr40^ axis may represent a critical event for the transition from ISC‐mediated homeostasis to CSC‐mediated tumorigenesis. At this stage, we have not yet observed aberrant crypt foci or adenoma in *Ctnnb1^T40D^* mutant mice, possibly due to the heterozygosity of the mutation. Whether homozygous mutation of *β*‐catenin T40D would cause autonomous colorectal adenoma warrants further investigation in the future.

Currently, the cell origin of CRC and CRC stem cell are not well understood. Our current work suggests cells expressing high levels of MST4 could represent an origin of CRC. Genetic tracing of MST4‐expressing cell lineage via *Stk26‐Cre;R26‐tdTomato* mice suggest a subpopulation of ISCs with high levels of MST4 might develop into colorectal cancer stem cells. It would also be intriguing to assess, potentially using an organoid system, the stemness of isolated CRC cells expressing high levels of MST4. In addition to characterizing the potential function of the MST4‐p*β*‐catenin^Thr40^ axis in CSCs, another unresolved but important question concerns the molecular mechanisms for dysregulation of this axis during CRC development.

Finally, it is worth mentioning that MST4 may phosphorylate multiple substrates, and could therefore exert in a context‐dependent manner complex influences and orchestrate different pathways during tumorigenesis. For example, we previously reported that MST4 targets the E3 ligase TRAF6 to limit innate inflammatory responses.^[^ ^43^ ^]^ Besides, our previous study observed that MST4 phosphorylates YAP and regulates Hippo/YAP signaling to suppress gastric tumorigenesis.^[^ ^44^ ^]^ Here, in the intestinal crypts of mice, we did not observe an apparent effect of MST4 on YAP under physiological condition; but did observe a positive role of MST4 on YAP protein levels during AOM/DSS‐induced tumorigenesis (data not shown). Thus, the regulatory function of MST4 appears to be highly context‐dependent. Different mice models could be useful for gaining overall insights into the pathological functions of MST4. In addition to the AOM/DSS and *Apc* mutation/knockout‐induced CRC mice models, we created here in this work a *Ctnnb1^T40D^* conditional knockin mice, which could mimic hyperactivation of *β*‐catenin and therefore possibly be used for modeling CRC.

In summary, this work uncovered an MST4‐p*β*‐catenin^Thr40^ axis required for accumulation of *β*‐catenin, and demonstrated an essential role for this axis in controlling ISCs/CSCs homeostasis and colorectal tumorigenesis.

## 4 Experimental Section

### Mouse Strains

*Mst4^floxed^* and *Mst4^T178E^* mice were generated using Cre/LoxP homologous recombination system with the principle, targeting strategies, and screening mechanisms briefly described in the previous study^[^ ^44^ ^]^ and Figure S6a in the Supporting Information. And, *Ctnnb1^T40D/+^* mice were obtained by homologous recombination system^[^ ^71^ ^]^ and the exon 2–15 of *Ctnnb1* was replaced by mutated exon 2–15 (T40D mutation at exon 3) by Shanghai Model Organisms Center (Shanghai, China). The experimental procedure and approaches were previously outlined.^[^ ^72^ ^]^ Chimeric mice were crossed with Flpe‐deleter mice^[^ ^71^ ^]^ to obtain *Mst4^floxed^* and *Mst4^T178E^* mice. *Villin‐Cre* mice were obtained from Shanghai Model Organisms Center (Shanghai, China). *Stk26‐Cre* mice were developed using Cre/LoxP recombination^[^ ^71^ ^]^ in Shanghai Model Organisms Center (Shanghai, China). *Apc^min/+^* mice^[^ ^66^ ^]^ were generated as described previously. *Lgr5‐EGFP‐ires‐CreERT2* mice,^[^ ^73^ ^]^ *Sox9‐CreERT2* mice,^[^ ^74^ ^]^ and *Apc^flox/+^* mice^[^ ^75^ ^]^ were kindly provided by Dr. Yi Arial Zeng (SIBCB, China), Dr. Bin Zhou (SIBCB, China), and Dr. Zhengquan Yu (China Agricultural University, China), respectively. All mice of both sexes were of a C57BL/6 background, and except where noted were 2 months old when subjected to the beginning of the experiments. The mice were cultured in automated watered and ventilated cages in a specific pathogen‐free environment on a 12 h light/dark cycle. All of the mice were randomly allotted to experimental groups in all experiments, which were conducted based on the guidelines of the Institutional Animal Care and Use Committee of the Institute of Biochemistry and Cell Biology.

The approval ID for animals breeding was SIBCB‐NAF‐14‐004‐S329‐023 issued by the Animal Core Facility of Center for Excellence in Molecular Cell Science (CAS), Chinese Academy of Sciences, University of Chinese Academy of Sciences, Shanghai, China.

### Immunofluorescence and Histopathology Analysis

The intestinal tracts were flushed with cold PBS, followed by 4% paraformaldehyde at 4 °C, and embedded in paraffin wax for sectioning. The sections (3 µm) were stained with indicated primary antibodies. The intestinal fractions were from at least four mice per group. Images were captured using laser confocal scanning microscopes equipped with either Olympus FV3000 (Olympus, Tokyo, Japan) 60× NA 1.36 oil immersion objective, or Zeiss 880 (Zeiss, Jena, Germany) 20× objective and 63× NA 1.4 oil immersion objective. Four lasers were used with wavelengths of 405, 488, 568, and 647 nm. Images were processed by using Imaris software and ImageJ software.

For detailed histologic analysis, sections were stained with hematoxylin & eosin (H&E) or periodic acid–Schiff,^[^ ^30^ ^]^ and analyzed with the aid of an Olympus BX51 dissecting microscope (Olympus, Tokyo, Japan).

### RNA In Situ Hybridization

The transcription of *Axin2* in intestinal tissues was detected in situ hybridization followed by the manufacturer's instructions (ACD, Hayward, CA, USA). Briefly, the tissue sections were deparaffinized, retrieved, and then hybridized with *Axin2* specific probes (ACD, Hayward, CA, USA). Then, the signal of hybridized probes was amplified using a cascade of signal amplification molecules and further visible by RED dye‐labeled probes. Subsequently, the sections were redyed with Gill II hematoxylin and mounted with VectaMount Permanent Mounting Medium (Vector Laboratories, Burlingame, CA, USA), and photographed using an Olympus BX53 photomicroscope (Olympus, Tokyo, Japan).

### Azoxymethane/dextran sodium sulfate‐induced murine colorectal cancer

An AOM‐DSS induced colorectal cancer in mice was generated as described previously with modifications.^[^ ^76^ ^]^ Mice at ages of about eight weeks were injected intraperitoneally with 10 mg kg^−1^ AOM (Sigma‐Aldrich, MO, USA). Then, the mice were treated with 1 g/100 mL DSS in drinking water (MP Biomedicals, Santa Ana, CA, USA) for 7 days after 7 days of AOM injection. After that, mice were given regular drinking water for 7 days, followed by three cycles (intraperitoneal injection of 10 mg kg^−1^ AOM and 7 days of 1% DSS) for *Sox9‐CreERT2;Mst4^T178E/T178E^* mice and *Sox9‐CreERT2;Ctnnb1^T40D/+^* mice; four cycles for *Sox9*‐CreERT2;*Mst4^fl/fl^* mice. At day 75 (*Sox9‐CreERT2;Mst4^T178E/T178E^* mice and *Sox9‐CreERT2;Ctnnb1^T40D/+^* mice) or at day 98 (*Sox9*‐CreERT2;*Mst4^fl/fl^* mice), the mice were sacrificed at the end of the model. There were at least four mice per group in the experiments. Section of colons were fixed in 4% paraformaldehyde and embedded in paraffin for histopathological analysis.

### Cell Culture

HEK293FT, SW480, MC38, and 3T3‐L1 cells (an immortalized mouse fibroblast cell line) were obtained from the Shanghai Life Academy of Sciences cell library (Shanghai, China). SW480 cells were cultured in L‐15 medium (Hyclone, Utah, USA) supplemented with 10% heat‐inactivated fetal bovine serum (FBS), 100 µg mL^−1^ penicillin, and 100 µg mL^−1^ streptomycin at 37 °C with no CO_2_ in a humidified incubator (Thermo, MA, USA); the other cells were grown in DMEM medium (Invitrogen, Carlsbad, CA) supplemented with 10% heat‐inactivated FBS, 100 µg mL^−1^ penicillin, and 100 µg mL^−1^ streptomycin at 37 °C with 5% CO_2_ in a humidified incubator (Thermo, MA, USA). All of the cells were obtained from the indicated sources and were passaged for less than 3 months. The identities of cell lines SW480 and MC38 were confirmed by carrying out fingerprinting (Shanghai Genening Biotechnologies Inc., Shanghai, China). The cells were regularly detected for mycoplasma using MycoAlert Mycoplasma Detection Kits (Lonza, ME, USA).

### Cell Proliferation and Sphere Formation

For cell proliferation assay, cells (2000 per well) were seeded in 96‐well plates after siMST4 RNAs transfection for 48 h. Then, cell numbers were measured and analyzed using an ATP‐based CellTiter‐Lumi Plus kit (Beyotime) based on the manufacturer's guidelines. The intracellular ATP contents were detected using a BioTek Synergy NEO multidetector microplate reader (Thermo). Cell growth was analyzed using the equation fold change = *N_t_*/*N*_0_, where *N_t_* is the value of ATP contents at time *t* h and *N*_0_ is the value of ATP contents at time 0 h.

For sphere formation assay, cells were suspended with DMEM/F12 medium (Gibco) with 100 µg mL^−1^ penicillin, 100 µg mL^−1^ streptomycin, 1× B‐27 (Invitrogen), 20 ng mL^−1^ basic FGF (Peprotech), and 20 ng mL^−1^ EGF (Peprotech) and seeded into 6‐well ultralow attachment plates (Corning, USA) for three repeats after siMST4 RNAs transfection for 48 h. At day 10, the number of sphere (area of sphere ≥70 µm^2^) was calculated and analyzed.

### Xenograft Tumor Model

Healthy male BALB/c nude mice were maintained in specific pathogen‐free conditions in available watered and ventilated cages on a 12 h light/dark cycle. During tumor formation, HCT116 cells (2 × 10^6^ cells per mice, *n* = 5 per group) and MC38 cells (5 × 10^5^ cells per mice, *n* = 4 per group) were respectively injected subcutaneously into BALB/c nude mice for at least four mice per group. After 4 days, the siRNAs were intratumorally injected every three days. The mice were sacrificed with CO_2_ after subsequent two weeks, and tumor volumes were then calculated as *V* = *L*_L_ × *L*_s_^2^/2 (*L*_L_, a length of longer diameter of tumor; and *L*_s_, a length of shorter diameter of tumor).

### Antibodies

Specific antibodies for Flag (M3165), *α*‐tubulin (T6199), and *β*‐actin (A2228) were acquired from Sigma (MO, USA). Those antibodies for Olfm4 (39141), *β*‐TrCP (4394), and HA (3724) were acquired from Cell Signaling Technology (MA, USA); those for Ki67 (ab15580), Lgr5 (ab75732), and lysosome (ab2408) were from Abcam (Cambridge, UK); those for *β*‐catenin (610153) were from BD Biosciences (San Jose, CA); those for SOX9 (AB5535) were from EMD Millipore (MA, USA); those for GSK3*α*/*β* (sc‐7291) and Laminin A/C (sc‐7292) were from Santa Cruz (Santa Cruz, CA); and those for MST4 and phospho‐*β*‐catenin (Thr40) were produced by Shanghai Immune Biotech (Shanghai, China). Secondary antibodies for Alexa Fluor 568 goat antirabbit IgG (H+L) (A11011), Alexa Fluor 647 donkey antigoat IgG (H+L) (A21447), and Alexa Fluor 488 goat antirabbit IgG (H+L) (A11001) were acquired from Invitrogen (Carlsbad, CA).

### Plasmids, siRNAs, and shRNAs

Flag‐MST4, its variants, and shMST4 were described previously.^[^ ^43^, ^44^ ^]^ *β*‐catenin and its variants (Flag‐tagged) were subcloned into a pCDH1 vector. The siRNAs targeting of MST4, *β*‐catenin, and the negative control was designed and synthesized by Genepharma (Shanghai, China). The sequences of these siRNAs were: for human siMST4‐1, the forward was 5′‐GCCUGAUCCAAAGAAAGUATT‐3′, and the reverse was 5′‐UACUUUCUUUGGAUCAGGCTT‐3′; for human siMST4‐2 targeting the UTR region of MST4, the forward was 5′‐AUUGUUACAAUAUAGGCACCATT‐3′, and the reverse was 5′‐UGGUGCCUAUAUUGUAACAAUTT‐3′; for mouse siMst4‐1, the forward was 5′‐CCAGCAGCAGGGAAAGUAATT‐3′, and the reverse was 5′‐UUACUUUCCCUGCUGCUGGTT‐3′; for mouse siMst4‐2, the forward was 5′‐GGGAAUUACUGCUAUUGAATT‐3′, and the reverse was 5′‐UUCAAUAGCAGUAAUUCCCTT‐3′; for human si*β*‐catenin‐1, the forward was 5′‐CUGGUUGUUAUGUGAUCAUTT‐3′, and the reverse was 5′‐AUGAUCACAUAACAACCAGTT‐3′; for human si*β*‐catenin‐2 targeting the UTR region of *β*‐catenin, the forward was 5′‐CCCUAGCCUUGCUUGUUAATT‐3′, and the reverse was 5′‐UUAACAAGCAAGGCUAGGGTT‐3′; for human si*β*‐catenin‐3 targeting the UTR region of *β*‐catenin, the forward was 5′‐GGGUAAAUCAGUAAGAGGUTT‐3′, and the reverse was 5′‐ACCUCUUACUGAUUUACCCTT‐3′; and for the negative control, the forward was 5′‐UUCUCCGAACGUGUCACGUTT‐3′, and the reverse was 5′‐ACGUGACACGUUCGGAGAATT‐3′.

### Knockout Cells Using CRISPR/Cas9 Method

Knockout cells were obtained according to the guidelines as previously described.^[^ ^77^ ^]^ Briefly, HEK293FT cells were transfected with lentiCRISPRv2‐sgRNAs plasmids for 48 h and selected using 2 µg mL^−1^ puromycin for 1 week. Then, knockout efficiency of the indicated proteins was detected using western blotting assay. MST4 sgRNAs were previously described.^[^ ^44^ ^]^ And CTNNB1 sgRNAs were designed based on human GeCKO library (https://www.genscript.com/CRISPR‐gRNA‐library.html). The sequences of CTNNB1 sgRNAs were: for human sgCTNNB1‐1, the forward was 5′‐GAAACAGCTCGTTGTACCGC‐3′, and the reverse was 5′‐GCGGTACAACGAGCTGTTTC‐3′; for human sgCTNNB1‐2, the forward was 5′‐GACACGCTATCATGCGTTCT‐3′, and the reverse was 5′‐AGAACGCATGATAGCGTGTC‐3′; for human sgCTNNB1‐3, the forward was 5′‐AATGCAGTTCGCCTTCACTA‐3′, and the reverse was 5′‐TAGTGAAGGCGAACTGCATT‐3′.

### Transfection and Luciferase Reporter Assay

siRNAs were transfected using Lipofectamine RNAiMAX Reagent (Invitrogen, San Diego, CA) with manufacturer's guidelines. And the plasmids were transfected by Lipofectamine 2000 (Invitrogen, San Diego, CA) based on the manufacturer's reference manual.

For luciferase reporter assay, HEK293FT cells or SW480 cells were transfected with TOP, FOP, Renilla, and indicated plasmids for 48 h for at least four repeats. Then, cells were lysed and the luciferase activities were measured using the Dual‐Luciferase Assay System (Promega).

### Real‐Time PCR

Total RNAs were extracted from cells using Total RNA Extraction Reagent (R401‐01‐AA, Vazyme), and then cDNA was reversed using the HiScriptII Q Select RT SuperMix (R223‐01, Vazyme) with the manufacturer's guidelines. Real‐time PCR was performed using Real‐time PCR Master Mix (YEASEN, Shanghai, China) according to an Applied Biosystems Step Two Real‐Time PCR System (Applied Biosystems, USA). *ACTB* or *Actb* was used as an internal control. The primers used were:

*hMST4*: 5′‐ATCTTGTGCAAACCCTGAGTTG‐3′ (F),
5′‐TTCAATCGCCTGATTCCTGCT‐3′ (R);
*hAXIN2*: 5′‐AGAAGCAGCAGATTGATTCC‐3′ (F),
5′‐CCCCCATTACTCATGTAAGC‐3′ (R);
*hCCND1*: 5′‐ CAATGACCCCGCACGATTTC ‐3′ (F),
5′‐ CATGGAGGGCGGATTGGAA ‐3′ (R);
*hACTB*: 5′‐ ATCATGAAGTGTGACGTGGA‐3′(F),
5′‐ CTCAGGAGGAGCAATGATCT‐3′(R);
*mMst4*: 5′‐TTTGGGAATTACTGCTATTGAACTT‐3′ (F),
5′‐TGGATGCATGTCAGAATTCG‐3′ (R);
*mAxin2*: 5′‐ATGAGTAGCGCCGTGTTAGTG‐3′ (F),
5′‐GGGCATAGGTTTGGTGGACT‐3′ (R);
*mCcnd1*: 5′‐GCGTACCCTGACACCAATCTC‐3′ (F),
5′‐CTCCTCTTCGCACTTCTGCTC‐3′ (R);
*mLgr5*: 5′‐CCTACTCGAAGACTTACCCAGT‐3′ (F),
5′‐GCATTGGGGTGAATGATAGCA‐3′ (R);
*mSox2*: 5′‐GCGGAGTGGAAACTTTTGTCC‐3′ (F),
5′‐CGGGAAGCGTGTACTTATCCTT‐3′ (R);
*mSox9*: 5′‐CAAGCGGAGGCCGAAGA‐3′ (F),
5′‐CAGCTTGCACGTCGGTTT‐3′ (R);
*mActb*: 5′‐ GGCTGTATTCCCCTCCATCG‐3′(F),
5′‐ CCAGTTGGTAACAATGCCATGT ‐3′(R).
Note: F, forward; R, reverse.

### Gene Expression RNA Sequence Analysis

For RNA sequence, samples were prepared from small intestinal epithelia of *Mst4^fl/fl^* mice and *Villin‐Cre;Mst4^fl/fl^* mice. Tissues of the small intestines were dissected into 2–5 mm^2^ fractions and placed into 1.5 mL Eppendorf tubes. They were incubated in Ca^2+^‐free, Mg^2+^‐free HEPES buffer containing 20 × 10^−3^ m EDTA and collagenase II at 37 °C for 30 min pulsed every 10 min with Vortex. The incubated tissues were subjected to low‐speed centrifugation, and IECs were collected from the centrifuged material and washed in cold HEPES (10 × 10^−3^ m) buffer. RNA was extracted from three biological replicates.

RNA quality was assessed using an Agilent Bioanalyzer 2100 (Agilent technologies, Santa Clara, CA, US) and sent for library preparation. Total RNA was amplified, labeled, and purified by RNAClean XP Kit (Beckman Coulter, Inc., Kraemer Boulevard Brea, CA, USA) and RNase‐Free DNase Set (QIAGEN, GmBH, Germany) following the manufacturer's guidelines. Then, the purified RNA was sequenced by Illumina Hiseq X10 platform by Majorbio Genomics (Shanghai, China), followed by analyzing the sequence data using GRCm38.p5 genome database.

The GEO accession number for the high‐throughput sequencing reported in this paper are GSE132565.

### Immunoprecipitation and Western Blotting

Whole cell contents were extracted from cells using western blotting buffer containing 20 × 10^−3^ m Tris·HCl, pH 8.0, 100 × 10^−3^ m NaCl, 0.5% Triton X‐100, and 1 × 10^−3^ m EDTA supplemented with Cocktail on shaking bed in 4 °C for 20 min. Then, the lysates were obtained by 4 °C centrifugation 13 000 rpm for 30 min and boiled with 5× SDS loading buffer. Finally, the proteins were separated by SDS‐PAGE, transfected to a PVDF membrane (Bio‐Rad), and blotted with indicated antibodies. Western blotting images were captured by the Mini Chemiluminescent Imaging and Analysis System (Beijing Sage Creation Science Co., Ltd.).

For immunoprecipitation assay, cells lysates were obtained using immunoprecipitation (IP) buffer containing 20 × 10^−3^ m Tris·HCl, pH 7.5, 100 × 10^−3^ m NaCl, 0.5%NP‐40, and 1 × 10^−3^ m EDTA supplemented with Cocktail. Then, the proteins were incubated with Flag‐beads (GenScript) or indicated antibodies together with protein A/G beads (Santa Cruz Biotechnologies) for overnight at 4 °C. And the beads were washed with IP buffer for three time and boiled with 1× SDS loading buffer. The interacted proteins were detected using indicated primary antibodies as stated above.

### Free *β*‐Catenin Extraction and Detection

Free *β*‐catenin was defined following previous literature (see main text), and experimentally prepared by using ProteoExract Native Membrane Protein Extraction Kit (#444810, Merck) as previously described^[^ ^78^ ^]^ according to the manufacturer's instructions. Briefly, the cytoplasmic contents except for membrane proteins were obtained using Extraction Buffers I. Then, cytoplasmic proteins containing free *β*‐catenin was boiled with 5× SDS loading buffer, and free *β*‐catenin was detected using *β*‐catenin antibodies.

### Protein Expression and Purification

His_6_‐tagged MST4 and *β*‐catenin or MBP‐tagged MST4 were cloned and expressed in pET28a vectors. Whole prokaryotic contents were induced and expressed by 0.5 × 10^−3^ m isopropyl‐*β*‐d‐thiogalactopyranoside (IPTG) in Terrific Broth medium at 16 °C in *Escherichia coli* BL21 (DE3) CodonPlus cells with the optical density of a sample at a wavelength of 600 nm (OD600) of 0.6–1.0. For His_6_‐tagged MST4 and *β*‐catenin proteins, the cells were collected by centrifugation after induction for 16 h and resuspended with lysis buffer A (20 × 10^−3^ m HEPES, pH 7.5, 500 × 10^−3^ m NaCl, 5% glycerol, 5 × 10^−3^ m *β*‐mercaptoethanol, and 20 × 10^−3^ m imidazole). Then, the cell lysates were obtained by a high‐pressure homogenizer and the supernatants were incubated with Ni Sepharose column (GE Healthcare) pre‐equilibrated with lysis buffer A. Finally, the proteins were eluted with lysis buffer A with 400 × 10^−3^ m imidazole. For MBP‐tagged MST4, the cells were resuspended in lysis buffer B (20 × 10^−3^ m HEPES pH 7.5, 500 × 10^−3^ m NaCl, 5% glycerol, 5 × 10^−3^ m *β*‐mercaptoethanol). And the contents were incubated to amylose resin (New England Biolabs) columns pre‐equilibrated with lysis buffer B. Then, the proteins were eluted with buffer B with 20 × 10^−3^ m maltose. The proteins were finally purified with size‐exclusion chromatography in an SEC buffer containing 20 × 10^−3^ m HEPES, pH 7.5, and 100 × 10^−3^ m NaCl for further experiments.

### Pulldown Assay

MBP‐tagged proteins coupled onto amylose resin beads were incubated with the indicated proteins in the buffer (20 × 10^−3^ m HEPES pH 7.5, 100 × 10^−3^ m NaCl, and 1 × 10^−3^ m dithiothreitol) at 4 °C for 1 h. Then, the beads were washed and eluted with the buffer adding 20 × 10^−3^ m maltose. The input and output samples were measured and analyzed with Coomassie Brilliant Blue staining or immunoblotting.

### In Vitro Kinase Assay

In Vitro kinase assay was performed according to the Kinase Reaction and Alkylation Protocol (abcam) as described previously.^[^ ^79^ ^]^ Briefly, purified MST4 and *β*‐catenin proteins were first co‐incubated in the buffer with 20 × 10^−3^ m HEPES pH 7.5, 100 × 10^−3^ m NaCl, 1 × 10^−3^ m ATP*γ*S, and 10 × 10^−3^ m MgCl_2_ for 30 min at 30 °C. Then, 2.5 × 10^−3^ m *p*‐nitrobenzyl mesylate (PNBM, ab138910) was added into the reaction system and incubated for 1 h at room temperature. The reaction mixture was detected using western blotting assay and the phosphorylated proteins were detected by a thiophosphate‐ester‐specific antibody (ab92570, Abcam, Cambridge, UK). In addition, Flag‐tagged *β*‐catenin (WT and mutants), GSK3*β*, and CK1*α* proteins were immunoprecipitated and purified from HEK293FT cells after being transfected with the indicated plasmids for 48 h using Flag beads. For in vitro kinase assay, purified *β*‐catenin proteins were first dephosphorylated as described previously,^[^ ^80^ ^]^ and then mixed with GSK3*β* and CK1*α* proteins in an ATP‐containing buffer, and further incubated for 30 min at 30 °C.

### Mass Spectrometry Assay

Purified MST4 and *β*‐catenin proteins were co‐incubated to induce phosphorylation of *β*‐catenin in the buffer containing 50 × 10^−3^ m Tris pH 7.5, 1 × 10^−3^ m ATP, 10 × 10^−3^ m MgAc2, and 0.1 × 10^−3^ m EGTA for 30 min at 30 °C. The reaction mixture was precipitated with TCA solution and subsequently dissolved in 8 m urea and 100 × 10^−3^ m Tris, pH 8.5. Then, mass spectrometry analysis was carried out in National Center for Protein Science Shanghai (Shanghai, China). For mass spectrometry (MS) assay of *β*‐catenin mutants, HEK293FT cells were transfected with the indicated plasmids and the cell lysates were immunoprecipitated using Flag‐beads. Then, the beads were washed for three times and resuspended in 8 m urea and 100 × 10^−3^ m Tris, pH 8.5. Finally, the mixture was measured and analyzed in Shanghai Majorbio Bio‐Pharm Technology Co., Ltd. (Shanghai, China).

### Ubiquitination Assay

The proteins of HA‐Ub (K48) and Flag‐*β*‐catenin mutants were coexpressed in HEK293FT cells for 48 h. Then, cytoplasmic contents were extracted from cells using IP buffer and incubated with HA antibodies and protein A/G beads (Santa Cruz Biotechnologies) for overnight at 4 ℃. Subsequently, the ubiquitination of *β*‐catenin was detected using western blotting assay with indicated primary antibodies.

### Human CRC Tissue Microarray

Tissue microarray sections were prepared by Outdo Biotech (Shanghai, China). All samples used were derived from 90 CRC patients approved by the Ethics Committee of Taizhou Hospital of Zhejiang province, China. Detailed clinical information was collected from all patients except 6, whose pathological records for TNM stage were lost. The median follow‐up period was 52 months (range, 3–102 months).

Tumor microarray sections were incubated with an anti‐MST4 antibody (1:100 dilution) or anti‐p‐*β*‐catenin (Thr40) antibody (0.35 mg mL^−1^, 1:25 dilution). Evaluation of immunohistochemical staining was carried out by two independent pathologists blinded to the medical records of patients. Results were assessed based on the extent (0, <5%; 1, 5–25%; 2, 26–50%; 3, 51–75%; 4, >75%) and intensity (0–3) of staining protein in 200 examined cells. Subsequently, the IHC result was calculated as 0–1, negative (−); 2–4, weakly positive (+); 5–8, moderately positive (++), and 9–12, strongly positive (+++).

### Statistical Analysis

Statistical analysis was performed using GraphPad Prism 8 (GraphPad Software, La Jolla, CA, USA) and SPSS statistical software ver. 13.0.1 (SPSS Inc., Chicago, IL). Normal distribution of continuous data was evaluated by the Kolmogorov–Smirnov test. The values were subjected to a standard normal distribution (*α* = 0.1), and then two‐tailed unpaired Student's *t*‐test was used for comparing between two groups. One‐way ANOVA and post hoc Bonferroni *t*‐test were performed for multiple comparisons. The n.s., *, **, and *** represent no significance, *p* > 0.05, *p* < 0.05, *p* < 0.01, and *p* < 0.001, respectively. Data represent means ± SD from two to three independent experiments. For transcription analysis, the data were acquired from at least three repeats. For quantitative analysis of immunofluorescence, the data were randomly captured and analyzed from murine intestinal tissues of at least four samples. For sequencing analysis, the mice were selected at random for three repeats to acquire RNA sequence data. The mice were selected at random for at least four samples to perform tumor number and size analysis of AOM/DSS‐induced mice model. The values of sample size were shown in figure legends and had no statistical methods to preprocess the exact values. The Fisher's Exact test was used to determine the interaction between two categorical variables. The correlation between two continuous variables was calculated by the Spearman's rank test. The Kaplan–Meier method and the log‐rank test were performed to analyze patients’ overall survival.

## Conflict of Interest

The authors declare no conflict of interest.

## Author Contributions

H.Z., M.L., and C.D. contributed equally to this work. H.Z. and S.J. performed most of the cellular experiments and in vivo analyses. M.C., Y.T., and W.W. made constructs for *β*‐catenin. M.C., F.W., J.J., L.H., and W.B. carried out biochemical analysis. M.L. and C.D. provided the CRC patient samples and performed related analyses. F.C. and M.W. carried out mice genotyping. D.W., L.L., Y.Z., S.X.H., X.L., J.L., and L.A. discussed and analyzed the data. H.Z., S.J., and Z.Z. designed the experiments and wrote the manuscript. Z.Z. and S.J. supervised the project.

## Supporting information

Supporting Information

## Data Availability

Research data are not shared.
