# Supplementary material for: An MST4‐pβ‐CateninThr40 Signaling Axis Controls Intestinal Stem Cell and Tumorigenesis
Source: Adv Sci (Weinh). 2021 Jul 8;8(17):2004850. doi: 10.1002/advs.202004850 (PMC8425901; doi:10.1002/advs.202004850)
Supplement: Supplementary file 1 — Supporting Information [file ADVS-8-2004850-s001.pdf]

© 2021 Wiley-VCH GmbH

ADVANCED SCIENCE
Open Access

Supporting Information

for *Adv. Sci.*, DOI: 10.1002/advs.202004850

An MST4-pβ-catenin^{Thr40} Signaling Axis Controls Intestinal Stem Cell and Tumorigenesis

Hui Zhang[1,2,9], Moubin Lin[3,9], Chao Dong[4,9], Yang Tang[5], Liwei An[5], Junyi Ju[5], Fuping Wen[5], Fan Chen[1], Meng Wang[2], Wenjia Wang[2], Min Chen[1], Yun Zhao[1], Jixi Li[2], Steven X. Hou[2], Xinhua Lin[2], Lulu Hu[6], Wenbo Bu[7], Dianqing Wu[8], Lin Li[1], Shi Jiao[2,5], Zhaocai Zhou[2*]*

Supporting Information

An MST4-pβ-catenin^{Thr40} Signaling Axis Controls Intestinal Stem Cell and Tumorigenesis

Supplementary Figure S1~S7

Supplementary Tables S1~S3

Supplementary Figures

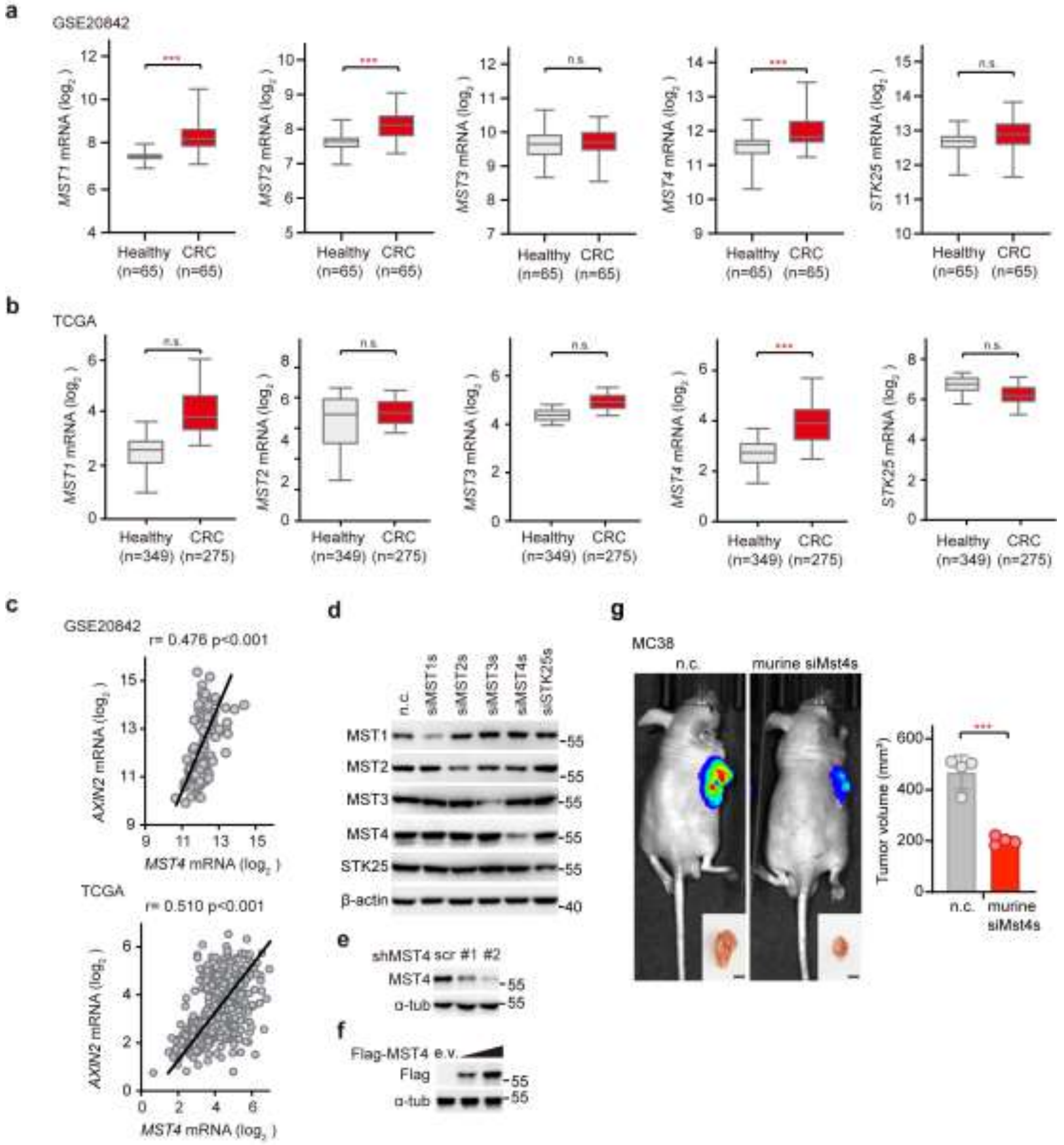

Figure S1. MST4 is up-regulated in CRC and depletion of MST4 inhibits tumor formation, Related to Figure 1. a,b) Transcription of *MST1*, *MST2*, *MST3*, *MST4* and *STK25* in CRC patients from the a) GEO (GSE20842) and b) TCGA. c) Positive correlation between *MST4* and *AXIN2* in CRC. mRNA levels of *MST4* were compared with those of *AXIN2* by Spearman's correlation. d) Immunoblotting to detect the expression of MST1, MST2, MST3, MST4 and STK25 in HEK293FT cells after transfected with the indicated siRNAs (two individual

siRNAs/gene). e,f) Immunoblotting of the indicated proteins in e) MST4-depleted and f) MST4-overexpressing HEK293FT cells. g) Xenograft tumor formation assay of MST4-depleted MC38 cells (n=4). Scale bar= 10 mm. murine siMST4s, the murine siRNAs mixture of siMST4#1 and siMST4#2. d,g) n.c., negative control. e) scr, scramble shRNA. f) e.v., empty vector. a,b,c,g) Data represent means ± SD. Two-tailed unpaired Student's *t* test for two variances, n.s., no significance, ***, $p < 0.001$.

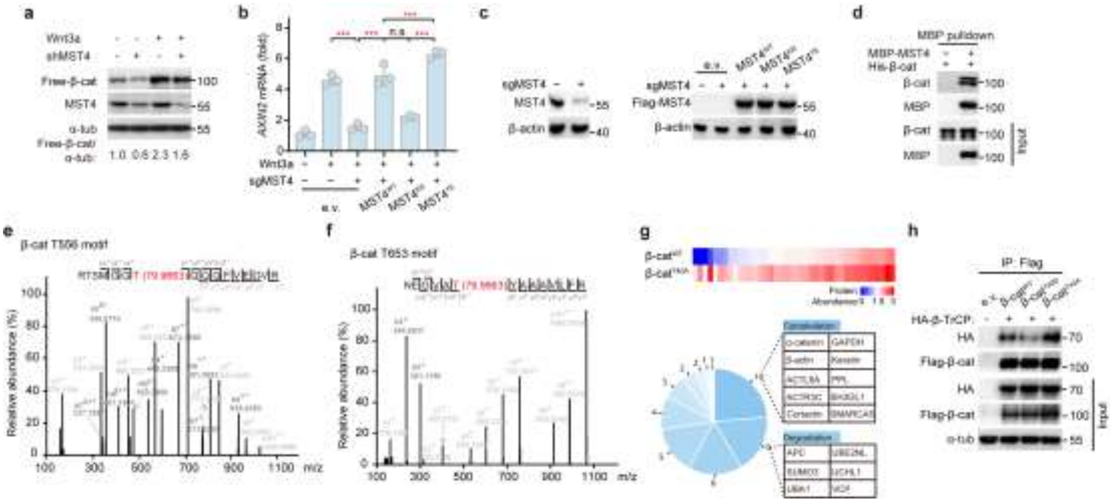

Figure S2. MST4 inhibits β-TrCP-mediated β-catenin degradation in a manner dependent on its kinase activity, Related to Figure 2. a) Immunoblotting of Free-β-cat in MST4-depleted cells. b) Rescue assay to detect *AXIN2* transcription in MST4-depleted HEK293FT cells after transfection with the indicated constructs following Wnt3a treatment. $MST4^{WT}$, wild type of MST4; $MST4^{TE}$, constitutively active mutant of MST4; $MST4^{KR}$, kinase dead mutant of MST4 (n=3). Data represent means ± SD. One-way ANOVA with post hoc Bonferroni *t* test for multiple variables comparison, n.s., no significance; ***, p<0.001. c) Immunoblotting to detect the indicated proteins. d) Pulldown assay to detect the interaction between MST4 and β-catenin. e,f) Mass spectrometry analysis of β-catenin phosphorylation by MST4 at e) Thr556 and f) Thr653. g) Heatmap (upper) and pie figure (lower) to indicate the interacting-proteins with β-catenin and its T40A mutant. h) Co-IP to analyze the interaction of β-TrCP with β-catenin and its mutants. b,c,h) e.v., empty vector.

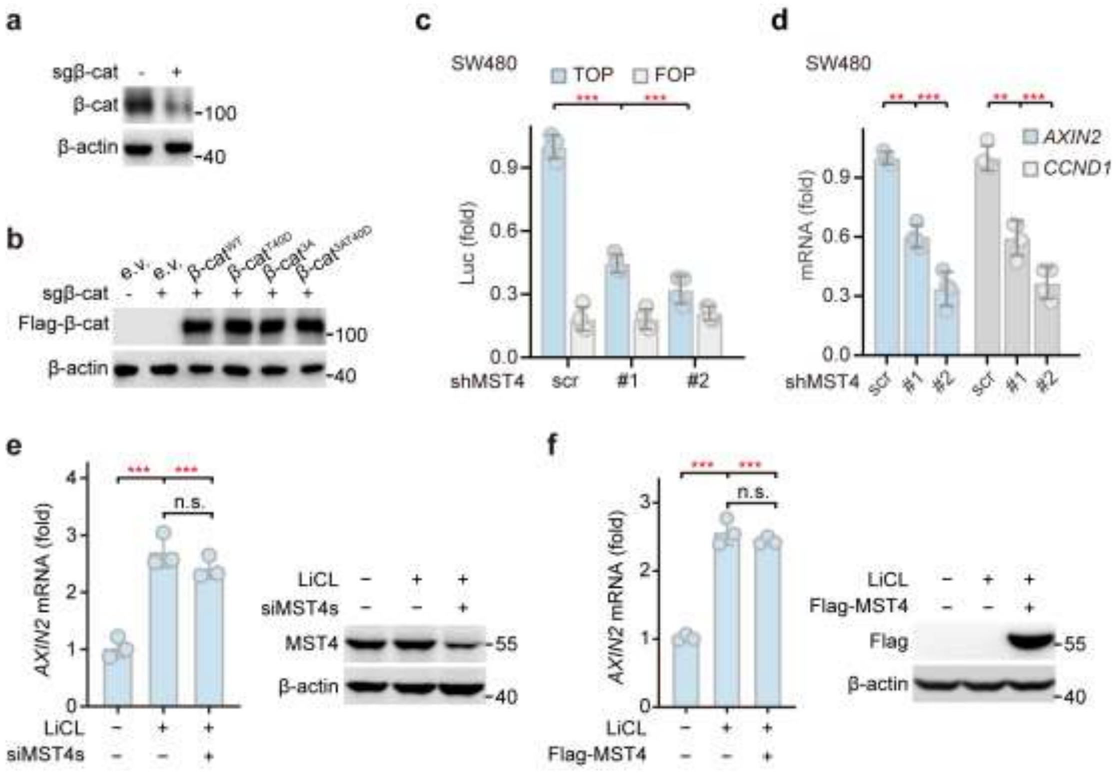

Figure S3. The regulatory effect of MST4 towards the transcription of Wnt target gene *AXIN2* in SW480 or HEK293FT cells, Related to Figure 3. a) Immunoblotting of β-catenin in β-cat-depleted HEK293FT cells. b) Immunoblotting of β-catenin in HEK293FT cells after transfection with the indicated plasmids. e.v. empty vector. c) TOP-FLASH activity in MST4-knockdown SW480 cells (n=4). d) mRNA levels of *AXIN2* and *CCND1* in MST4-knockdown SW480 cells (n=4). e) Transcription of *AXIN2* in MST4-depleted HEK293FT cells following LiCL treatment (n=3). Immunoblotting to indicate the knockdown efficiency of siMST4s. f) mRNA levels of *AXIN2* in MST4-overexpressing HEK293FT cells following LiCL treatment (n=3). Immunoblotting to indicate the expression of MST4. c,d) scramble shRNA. c-f) Data represent means ± SD. One-way ANOVA with post hoc Bonferroni *t* test for comparing multiple variables, **, p<0.01; ***, p < 0.001; n.s., no significance.

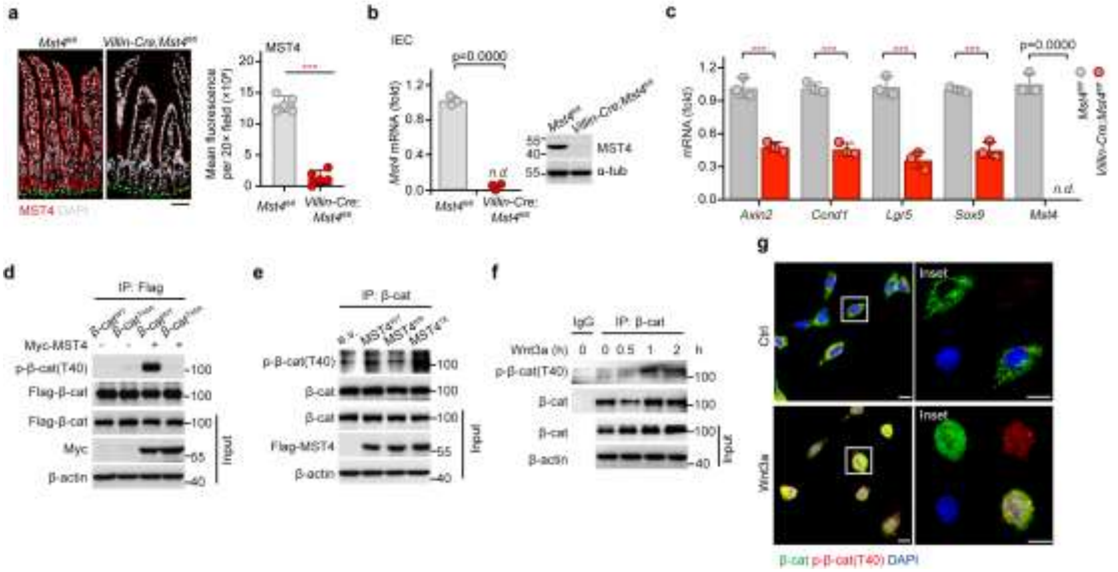

Figure S4. MST4 phosphorylates β-catenin at Thr40 in response to Wnt3a stimulation, Related to Figure 4. a) Immunofluorescent staining of MST4 in the small intestines derived from *Mst4$^{fl/fl}$* and *Villin-Cre;Mst4$^{fl/fl}$* mice (n=5). b) Knockout efficiency of MST4 in the small intestines of *Mst4$^{fl/fl}$* and *Villin-Cre;Mst4$^{fl/fl}$* mice analyzed using real-time RT-PCR and immunoblotting (n = 3). c) mRNA levels of the indicated Wnt target genes (*Axin2*, *Ccnd1*, *Lgr5*, and *Sox9*) in *Mst4$^{fl/fl}$* and *Villin-Cre;Mst4$^{fl/fl}$* mice (n=3). d) Immunoblotting of p-β-cat (T40) in cells after transfection with wild type β-catenin and its T40A mutants. e) Immunoblotting of p-β-cat (T40) in cells after transfection with MST4 and its mutants. f,g) Immunoblotting f) and immunofluorescence g) of p-β-cat (T40) in cells after Wnt3a treatment. Ctrl, control group. a,g) Scale bar, 10 μm. a-c) Data represent means ± SD. Two-tailed unpaired Student's *t* test for two variances, ***, p<0.001.

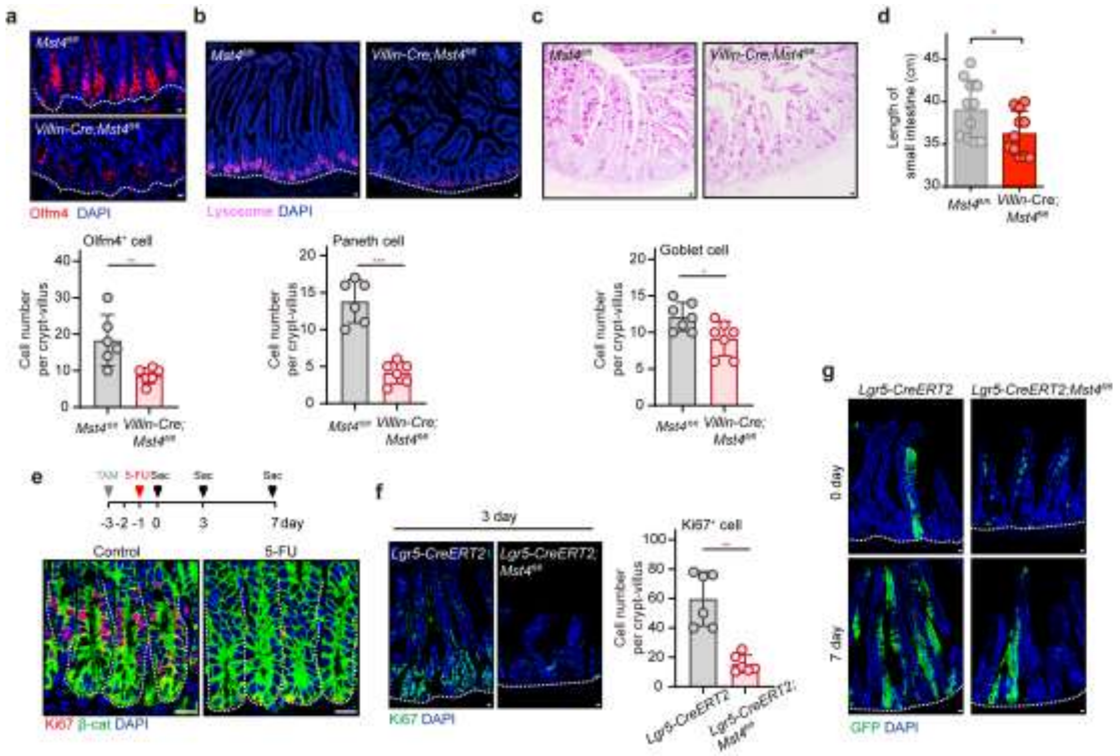

Figure S5. Depletion of MST4 disrupts intestinal homeostasis, Related to Figure 5. a) Immunofluorescent analysis of Olfm4^{+} cells in crypts from *Mst4$^{fl/fl}$* and *Villin-Cre;Mst4$^{fl/fl}$* mice (n=6). b) Fluorescent staining of lysosome in small intestine sections derived from the indicated mice (n=6). Lysosome, paneth cell marker. c) PAS staining of small intestine sections derived from the indicated mice (n=7). PAS staining for goblet cells. d) Average length of the small intestines in *Mst4$^{fl/fl}$* and *Villin-Cre;Mst4$^{fl/fl}$* mice (n=11). e) Immunofluorescent staining of Ki67 and β-catenin in crypts derived from the mice after 5-FU treatment. f) Immunofluorescent analysis of Ki67 in small intestines after 5-FU treatment. g) Accelerated expansion of Lgr5 initiated lineage tracing. GFP images in the small intestine from the indicated mice. a-c,e-g) Scale bar, 10 µm. a-d,f) Data represent means ± SD. Two-tailed unpaired Student's *t* test for two variances, *, p<0.05; **, p<0.01; ***, p<0.001.

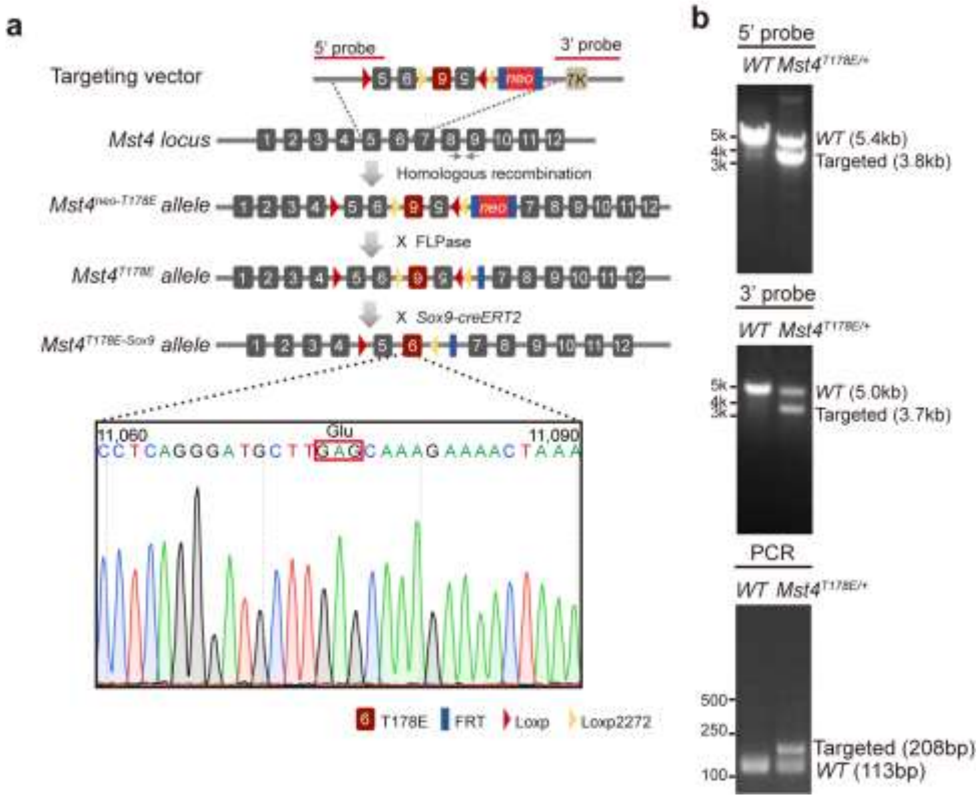

Figure S6. Generation and verification of the *Mst4*^{T178E} allele, Related to Figure 6. a) Generation and verification of the *Mst4*^{T178E} allele. b) Genotyping of *Mst4*$^{T178E/+}$ mice.

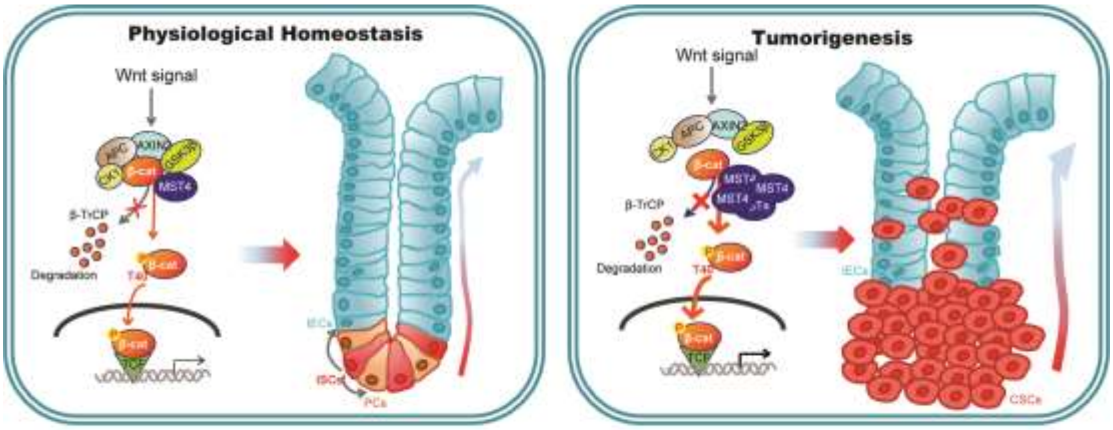

Figure S7. A working model for the MST4-pβ-catenin^{Thr40} signaling axis regulating intestinal homeostasis and CRC development, Related to Figures 1-8. According to this model, MST4 directly phosphorylates β-catenin at T40 to completely inhibit phosphor-Ser33 of β-catenin by GSK3β thus to block its interaction with β-TrCP, thus impeding the degradation of β-catenin and playing a key role during intestinal tissue homeostasis. During colorectal tumorigenesis, hyperactivation of MST4-pβ-catenin^{Thr40} axis promotes CRC progression.

Supplementary Tables

Table S1 Clinical Significance of MST4 Levels in CRC Progression

Groups	MST4 expression				n	Positive (%)	p value (*Fisher's test*)
	−	+	+ +	+ + +			
Sex							
Male	10	18	10	16	54	81.5	0.1104
Female	4	4	6	16	30	86.7	
Age							
<65	6	12	9	13	40	85.0	0.6616
>=65	8	10	7	19	44	81.8	
Tumor Size							
T1/2	7	6	2	3	17	64.7	0.0158*
T3/4	7	16	14	29	67	88.1	
Lymph node metastasis							
N0	9	15	4	14	42	78.6	0.0365*
N1/2	5	7	12	18	42	88.1	
Distant metastasis							
M0	12	16	13	31	72	83.3	0.0541
M1	2	6	3	1	12	83.3	
Tumor stage							
I/II	8	13	10	8	39	79.5	0.0205*
III/IV	6	9	6	24	45	86.7	
Total	14	22	16	32	84		

Note: Fisher's exact test was used to test the association between two categorical variables;
* represents statistically significant, p<0.05.

Table S2 Clinical Significance of β-catenin (T40) Phosphorylation in CRC Progression

Groups	β-catenin (T40) Phosphorylation				n	Positive (%)	p value
	**−**	**+**	**+ +**	**+ + +**			(*Fisher's test*)
Sex							
Male	10	13	15	16	54	81.5	0.500
Female	4	4	9	13	30	86.7	
Age							
<65	8	9	12	11	40	80.0	0.611
>=65	6	8	12	18	44	86.4	
Tumor Size							
T1/2	6	6	3	2	17	64.7	0.046*
T3/4	8	11	21	27	67	88.1	
Lymph node metastasis							
N0	10	12	10	10	42	76.2	0.035*
N1/2	4	5	14	19	42	90.5	
Distant metastasis							
M0	14	16	22	20	72	80.6	0.069
M1	0	1	2	9	12	100.0	
Tumor stage							
I/II	10	11	8	10	39	74.4	0.029*
III/IV	4	6	16	19	45	91.1	
Total	14	17	24	29	84		

Note: Fisher's exact test was used to test the association between two categorical variables;
* represents statistically significant, $p<0.05$.

Table S3 Clinical association of β-catenin (T40) Phosphorylation and MST4 levels in CRC

Groups	β-catenin (T40) Phosphorylation				n	p value (*Fisher's test*)
	**−**	**+**	**+ +**	**+ + +**		
MST4 level						
**−**	4	5	3	2	14	0.011*
**+**	4	7	4	7	22	
**+ +**	3	3	8	2	16	
**+ + +**	3	2	9	18	32	
Total	14	17	24	29	84	

Note: Fisher's exact test was used to test the association between two categorical variables; * represents statistically significant, $p<0.05$.
